# Deep Learning for 3D Reconstruction, Augmentation, and Registration: A Review Paper

**DOI:** 10.3390/e26030235

**Published:** 2024-03-07

**Authors:** Prasoon Kumar Vinodkumar, Dogus Karabulut, Egils Avots, Cagri Ozcinar, Gholamreza Anbarjafari

**Affiliations:** 1 iCV Lab, Institute of Technology, University of Tartu, 50090 Tartu, Estonia; prasoon.vinodkumar@ut.ee (P.K.V.); dogus.karabulut@ut.ee (D.K.); chagri.ozchinar@ut.ee (C.O.); 2 PwC Advisory, 00180 Helsinki, Finland; 3 iVCV OÜ, 51011 Tartu, Estonia; 4 Institute of Higher Education, Yildiz Technical University, Beşiktaş, Istanbul 34349, Turkey

**Keywords:** deep learning, 3D reconstruction, 3D augmentation, 3D registration, point cloud, voxel, neural networks, convolutional neural networks, graph neural networks, generative adversarial networks, review

## Abstract

The research groups in computer vision, graphics, and machine learning have dedicated a substantial amount of attention to the areas of 3D object reconstruction, augmentation, and registration. Deep learning is the predominant method used in artificial intelligence for addressing computer vision challenges. However, deep learning on three-dimensional data presents distinct obstacles and is now in its nascent phase. There have been significant advancements in deep learning specifically for three-dimensional data, offering a range of ways to address these issues. This study offers a comprehensive examination of the latest advancements in deep learning methodologies. We examine many benchmark models for the tasks of 3D object registration, augmentation, and reconstruction. We thoroughly analyse their architectures, advantages, and constraints. In summary, this report provides a comprehensive overview of recent advancements in three-dimensional deep learning and highlights unresolved research areas that will need to be addressed in the future.

## 1. Introduction

Autonomous navigation, domestic robots, the reconstruction of architectural models of buildings, facial recognition, the preservation of endangered historical monuments, the creation of virtual environments for the film and video game industries, and augmented/virtual reality are just a few examples of real-world applications that depend heavily on the identification of 3D objects based on point clouds. A rising number of these applications require three-dimensional (3D) data. Processing 3D data reliably and effectively is critical for these applications. A powerful method for overcoming these obstacles is deep learning. In this review paper, we concentrate on deep learning methods for *reconstruction*, *augmentation*, and *registration* in three dimensions.

The processing of 3D data employs a wide range of strategies to deal with unique problems. *Registration*, which entails matching several point clouds to a single coordinate system, is one key issue. While conventional approaches rely on geometric changes and parameter optimisation, deep learning provides an all-encompassing approach with promising outcomes. *Augmentation* is another technique for deep learning employed in 3D data processing, and it entails transforming current data while maintaining the integrity of the underlying information to produce new data. Since augmentation may provide new data points that enhance the accuracy and quality of the data, it is a useful technique for resolving problems with data quality and completeness. The final technique in this analysis is called *reconstruction*, which entails building a 3D model from a collection of 2D photos or a 3D point cloud. This is a difficult task since 3D geometry is complicated and 3D data lack spatial order. In order to increase the accuracy and effectiveness of reconstruction, deep learning algorithms have made substantial advancements in this field by proposing novel architectures and loss functions. Overall, these methods have shown promise in resolving the difficulties involved in interpreting 3D data and enhancing the accuracy and value of 3D data.

### 1.1. Our Previous Work

We have previously conducted [1] an in-depth review of recent advancements in deep learning approaches for 3D object identification, including 3D object segmentation, detection, and classification methods. The models covered in our earlier article were selected based on a number of factors, including the datasets on which they were trained and/or assessed, the category of methods to which they belong, and the tasks they carry out, such as segmentation and classification. The majority of the models that we surveyed in our earlier study were validated, and their results were compared with state-of-the-art technologies using benchmark datasets such as SemanticKITTI [2] and Stanford 3D Large-Scale Indoor Spaces (S3DIS) [3]. We discussed in detail some of the most advanced and/or benchmarking deep learning methods for 3D object recognition in our earlier work. These methods covered a range of 3D data formats, such as RGB-D (IMVoteNet) [4], voxels (VoxelNet) [5], point clouds (PointRCNN) [6], mesh (MeshCNN) [7], and 3D video (Meta-RangeSeg) [1,8].

### 1.2. Research Methodology

In this paper, we provide a comprehensive overview of recent advances in deep-learning-based 3D object reconstruction, registration, and augmentation as a follow-up to our earlier research [1]. It concentrates on examining frequently employed building components, convolution kernels, and full architectures, highlighting the benefits and drawbacks of each model. Over 37 representative papers that include 32 benchmark and state-of-the-art models and five benchmark datasets that have been used by many models over the last five years are included in this study. Additionally, we review six benchmark models related to point cloud completion over the last five years. We selected these papers based on the number of citations and implementations by other researchers in this field of study. Despite the fact that certain notable 3D object recognition and reconstruction surveys, such as those on RGB-D semantic segmentation and 3D object reconstruction, have been published, these studies do not exhaustively cover all 3D data types and common application domains. Most importantly, these surveys only provide a general overview of 3D object recognition techniques, including some of their advantages and limitations. The current developments in these machine learning models and their potential to enhance the accuracy, speed, and effectiveness of 3D registration, augmentation, and reconstruction are the main reasons for our selection of these particular models. In real-world situations, the use of many of these models in a pipeline has the potential to improve performance even more significantly and achieve even better outcomes.

## 2. 3D Data Representations

### 2.1. Point Clouds

Raw 3D data representations, like point clouds, can be obtained using many scanning technologies, such as Microsoft Kinect, structured light scanning, and many more. Point clouds have their origins in photogrammetry and, more recently, in LiDAR. A collection of randomly arranged points in three dimensions, known as a point cloud, resembles the geometry of three-dimensional objects. The implementation of these points results in a non-Euclidean geometric data format. A further way to describe point clouds is to describe a collection of small Euclidean subsets with a common coordinate system, global parametrisation, and consistency in translation and rotation. As a result, determining the structure of point clouds depends on whether the object’s global or local structure is taken into account. A point cloud can be used for a range of computer vision applications, including classification and segmentation, object identification, reconstruction, etc. It is conceptualised as a collection of unstructured 3D points that describe the geometry of a 3D object.

Such 3D point clouds can be easily acquired, but processing them can be challenging. Applying deep learning to 3D point cloud data is riddled with difficulties. These issues include point alignment issues, noise/outliers (unintended points), and occlusion (due to congregated scenery or blindsides). Table 1 provides the list of 3D reconstruction models using point cloud representation reviewed in this study. The following, however, are the most significant challenges in applying deep learning to point clouds:

*Irregular*: Depending on how evenly the points are sampled over the various regions of an object or scene, point cloud data may include dense or sparse points in different parts of an item or scene. Techniques for subsampling can minimise irregularity, but they cannot get rid of it entirely.

*Unordered*: The collection of points acquired around the objects in a scene is called a point cloud, and it is frequently preserved as a list in a file. These points are earned by interacting with the objects in the scenario. The set itself is referred to as being permutation-invariant since the scene being shown remains constant regardless of the order in which the points are arranged.

*Unstructured*: A point cloud’s data are not arranged on a conventional grid. The distance between each point and its neighbours is individually scanned; therefore, it is not always constant. The space between two adjacent pixels in a picture, on the other hand, remains constant and can only be represented by a two-dimensional grid.

### 2.2. Voxels

Using three-dimensional volumes is an alternative way of representing three-dimensional surfaces using a grid of constant size and dimensions. Three-dimensional data can be represented as a regular grid in three-dimensional space. Voxels are a three-dimensional data description method that defines how an object in three-dimensional space is spread across all three dimensions of a scene. Voxels are used to model 3D data by defining the distribution of the 3D object across the scene’s three dimensions. By identifying the occupied voxels as visible, occluded, or self-occluded, viewpoint information about the 3D shape may also be conveyed. Encoding the view information for a 3D shape enables the occupied voxels to be classified as either visible blocks or self-occluded voxels. These grids are maintained either as a binary occupancy grid, where the cell values represent the voxel occupancy, or as a signed distance field, where the voxels represent the distances to the zero-level set that represents the surface boundary. The binary occupancy grid is the more prevalent storage format of the two. Table 2 provides the list of 3D reconstruction models using voxel representation reviewed in this study.

Despite the simplicity of the voxel-based representation and its capacity to encode information about the 3D shape and its viewpoint, it is constrained by one main constraint:

*Inefficient*: The inefficiency of voxel-based representation stems from the fact that it represents both occupied and unoccupied portions of a scene, which creates an excessive need for memory storage. This is why voxel-based representations are unsuitable for high-resolution data representation.

### 2.3. Meshes

3D meshes are one of the most commonly used ways to represent 3D shapes. A 3D mesh structure is composed of a set of polygons called faces, which are represented in terms of a set of vertices that describe the mesh’s coordinates in 3D space. The connection list associated with these vertices describes how they are connected to one another. Following the grid-structured data, the local geometry of the meshes can be described as a subset of Euclidean space. Table 3 provides the list of 3D reconstruction models using mesh representation reviewed in this study.

Meshes are non-Euclidean data where the known properties of the Euclidean space, such as shift-invariance, operations of the vector space, and the global parametrisation system, are not well defined. Learning from 3D meshes is difficult for two key reasons:

*Irregular*: Deep learning approaches have not been effectively extended to such irregular representations, and 3D meshes are highly complex.

**Figure 1:**
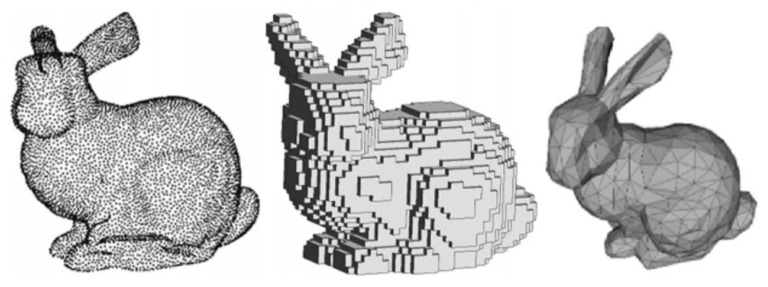
The 3D data representations of the Stanford Bunny [33] model: point cloud (**left**), voxels (**middle**), and 3D mesh (**right**) [34].

*Low quality*: In addition, such data typically contain noise, missing data, and resolution issues. Figure 1 shows 3D data representations of the Stanford Bunny [33] dataset with point cloud, voxel, and mesh data representations [34].

**Table 3:** 3D reconstruction models using mesh data representation.

Model	Dataset	Data Representation
Neural renderer [35]	ShapeNet [10]	Meshes
Residual MeshNet [36]	ShapeNet [10]	Meshes
Pixel2Mesh [37]	ShapeNet [10]	Meshes
CoReNet [38]	ShapeNet [10]	Meshes

## 3. 3D Benchmark Datasets

The datasets used in deep learning for 3D registration, augmentation, and reconstruction significantly influence the model’s accuracy and effectiveness. In order to train and assess deep learning models for 3D registration, augmentation, and reconstruction, it is imperative to have access to a wider variety of representative datasets. Future studies should concentrate on creating larger and more realistic datasets that include a variety of real-world objects and environments. For 3D registration, augmentation, and reconstruction, this would make it possible to develop even deeper learning models that are more reliable and accurate. This article will only list the most common datasets that have been used by the 3D object registration, augmentation, and reconstruction models discussed in this survey paper in Section 3 (3D reconstruction), Section 4 (3D registration), and Section 5 (3D augmentation). This includes the ModelNet [28], PASCAL3D+ [22], ShapeNet [10], ObjectNet3D [14] and ScanNet [39] datasets. Datasets that are specific only to some 3D recognition models will not be included in this survey. Table 4 provides the properties of data provided by different datasets.

### 3.1. ModelNet

By combining 3D CAD models from 3D Warehouse, 261 CAD model websites indexed with the Yobi3D search engine, common item categories searched from the SUN database [25], models from the Princeton Shape Benchmark [40], and models from the SUN database that contain at least 20 object instances per category, ModelNet [28] is a large-scale object collection of 3D computer graphics CAD models. Both the total number of categories and the total number of occurrences per category were constrained in a number of earlier CAD datasets. The writers thoroughly examined each 3D model and removed extraneous elements from each CAD model, such as the floor and thumbnail images, such that each mesh model had just one item from the designated category. ModelNet is almost 22 times larger than the Princeton Shape Benchmark [40] which contains 151,128 3D CAD models representing 660 distinct item categories. ModelNet10 and ModelNet40 are mostly used for classifying and recognising objects.

### 3.2. PASCAL3D+

Each of the 12 categories of 3D stiff objects that can be found in PASCAL3D+ [22] contains more than 3000 individual items. Pose estimation and the detection of 3D objects are also possible applications for the dataset. In addition to that, it might function as a baseline for the community. Images from PAS-CAL show a lot more diversity and more closely resemble actual situations. As a result, this dataset is less skewed than those that are gathered in controlled environments. Viewpoint annotations are continuous and dense in this dataset. The perspective is usually discretised into numerous bins in the current 3D datasets. Consequently, detectors that have been trained on this dataset may be more broadly capable. The objects in this collection are truncated and occluded; such objects are typically disregarded in the 3D datasets available today. Three-dimensional annotations are added to 12 rigid categories in the PASCAL VOC 2012 [41] dataset using PASCAL3D+. A selection of CAD models that cover intra-class variability are downloaded for each category. The closest CAD model in terms of 3D geometry is then linked to each occurrence of an object inside the category. Additionally, a number of 3D landmarks inside these CAD models have been discovered, and annotators have labelled the landmarks’ 2D positions. Eventually, an accurate continuous 3D posture for each item in the collection is generated utilising the 3D–2D correspondences of the landmarks. Consequently, the CAD model that corresponds with each item, along with 2D landmarks and the 3D continuous position, makes up its annotation.

### 3.3. ShapeNet

More than 50,000 CAD models are available in ShapeNet [10], a significant collection of shapes organised into 55 categories. Additionally, there are annotations for semantic features and categories. This large dataset consists of semantic category labels for models, rigid alignments, parts, bilateral symmetry planes, physical sizes, and keywords, in addition to further recommended annotations. ShapeNet had over 3 million models indexed when the dataset was released, and 220,000 models had been categorised into 3140 categories. ShapeNetCore is a subset of ShapeNet, which has over 51,300 unique 3D models. There are annotations for 55 common item categories. ShapeNetSem is a subset of ShapeNet, which includes 12,000 models. It is more condensed yet has 270 more thorough categories. By making ShapeNet the first large-scale 3D shape dataset of its sort, it has advanced computer graphics research in the direction of data-driven research, building on recent advancements in vision and NLP. It has also supported a wide class of newly revived machine learning and neural network approaches for applications dealing with geometric data by offering a large-scale, extensively annotated dataset.

### 3.4. ObjectNet3D

Despite having 30,899 photos, PASCAL3D+ [22] is still unable to fully capture the variances of common item categories and their geometric variety due to its limitation in the number of object classes (12 total) and 3D forms (79 total). A large-scale 3D object collection with more item categories, more 3D forms per class, and precise image-shape correspondences is provided by ObjectNet3D [14]. This dataset is comprised of a total of 90,127 photos in 100 distinct categories. Annotations pertaining to the 3D posture as well as the shape of each 2D object found in the photographs are provided. It is also useful for problems involving the development of proposals, the detection of objects in two dimensions, and the estimation of poses in three dimensions. For the automotive category, for instance, 3D forms of sedans, SUVs, vans, trucks, etc., are provided. The sizes of these three-dimensional forms have been normalised to fit [1] within a unit sphere, and they have been oriented in accordance with the category’s primary axis (e.g., front view of a bench). Additionally, each 3D form has a set of personally chosen keypoints that may be used to identify significant points in photos or 3D shapes. In total, 783 3D shapes from all 100 categories have been gathered in this manner.

### 3.5. ScanNet

**Figure 2:**
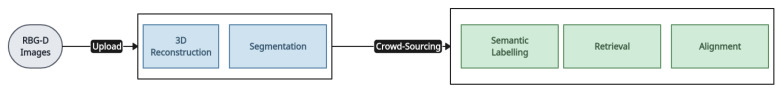
RBG-D reconstruction and semantic annotation framework of ScanNet [39] dataset.

ScanNet [39] is a collection of RGB-D scans of real-world locations with extensive annotations. It contains 2.5 million RGB-D pictures from 1513 scans taken in 707 different settings. Due to its annotation with approximated calibration parameters, camera postures, 3D surface reconstructions, textured meshes, dense object-level semantic segmentations, and aligned CAD models, the scope of this research is substantial. A capture pipeline is created to make it simpler for novices to obtain semantically labelled 3D models of situations in order to establish a framework that enables many individuals to gather and annotate enormous amounts of data. Data are collected, and off-line processing is performed on RGB-D video. The scene is completely 3D reconstructed and semantically labelled. With the use of ScanNet data, 3D deep networks can be trained, and their performance on a variety of scene comprehension tasks, such as 3D object categorisation, semantic voxel labelling, and CAD model retrieval, can be assessed. ScanNet has several different kinds of places, including offices, homes, and bathrooms. A versatile framework for RGB-D acquisition and semantic annotations is offered by ScanNet. Cutting-edge performance on a number of 3D scene interpretation tasks is made possible with the support of ScanNet’s fully annotated scan data. Finally, crowdsourcing employing semantic annotation tasks is used to collect instance-level item category annotations and 3D CAD model alignments for reconstruction. The RBG-D reconstruction and semantic annotation framework is shown in Figure 2.

Similar to our previous work [1], to determine which model performs better with each of these datasets, we attempted to compare the performance of the models that use them. While some of the models analysed in this study concentrate on computation time (measured in milliseconds), others focus on performance metrics like accuracy and precision. The majority of these models have assessed their efficacy using visual shape identification of the objects rather than numerical values. As a result, we were unable to compare the performance of these models using the datasets provided.

## 4. Object Reconstruction

Two types of traditional 3D reconstruction techniques exist: model-driven and data-driven techniques. The goal of the model-driven approaches is to align the item types in a library with the geometry of the objects created using digital surface models (DSMs), such as point clouds [42]. By using this method, the topological correctness of the rebuilt model can be guaranteed; nevertheless, issues might arise if the object shape has no candidates in the library. Additionally, the production accuracy is decreased by model-driven procedures since they only use a small fraction of the pre-defined shapes that are provided in the model libraries. Furthermore, modelling complicated object structures might not be possible. A DSM (often in the form of a point cloud) is used as the main data source in data-driven approaches, and the models are created from these data overall, without focusing on any one parameter. The primary issue with the data-driven technique is the possibility of unsuccessful segment extraction, which could result in topological or geometrical errors throughout the intersection process. Typically, data-driven techniques lack robustness and are extremely susceptible to data noise. Because data-driven methods are sensitive to noise, pre-processing data is a crucial step in preventing inaccurate outcomes [43].

### 4.1. Procedural-Based Approaches

The extensive and demanding field of automated reconstruction of 3D models from point clouds has attracted significant attention in the fields of photogrammetry, computer vision, and computer graphics due to its potential applications in various domains, including construction management, emergency response, and location-based services [44]. However, the intrinsic noise and incompleteness of the data provide a hurdle to the automated construction of the 3D models and necessitate additional research. These methods extract 3D geometries of structures, such as buildings, solely through a data-driven process that is highly dependent on the quality of the data [45,46].

Procedural-based techniques use shape grammars to reconstruct interior spaces while taking advantage of architectural design principles and structural organisation [47,48]. Because these methods take advantage of the regularity and recurrence of structural parts and architectural design principles in the reconstruction, they are more resilient to data incompleteness and uncertainty. Shape grammars are widely and successfully utilised in the field of urban reconstruction for 3D synthesising architecture (e.g., building façades) [49]. This procedural-based strategy is less sensitive to inaccurate and partial data than the data-driven alternatives. Several academics have successfully proposed shape grammars based on integration with a data-driven method to procedurally recreate building façade models from observation data (i.e., photos and point clouds) in order to reconstruct models of real settings [50,51].

However, because indoor and outdoor contexts differ from one another, the façade grammars cannot be used directly there. The translation of architectural design knowledge and principles into a grammar form, which guarantees the topological accuracy of the rebuilt elements and the plausibility of the entire model, is generally where shape-grammar-based systems have their advantages [44]. A set of grammar rules is necessary for procedural-based approaches, and in the grammar-based indoor modelling techniques currently in use, the parameters and rule application sequence are manually specified. However, these techniques are frequently restricted to straightforward architectural designs, such as the Manhattan design [48,52].

### 4.2. Deep-Learning-Based Approaches

Artificial intelligence (AI) is profoundly altering the way the geographical domain functions [53]. There is hope that the constraints of traditional 3D modelling and reconstruction techniques can be solved by the recently established deep learning (DL) technologies. In recent years, there has been a lot of study on 3D reconstruction using deep learning, with numerous articles covering the subject. Comparing the DL approaches to the traditional methods, state-of-the-art results were obtained [54,55,56]. With the recent rapid growth in 3D building models and the availability of a wide variety of 3D shapes, DL-based 3D reconstruction has become increasingly practical. It is possible to train DL models to recognise 3D shapes and all of their attributes [43].

Computational models with several processing layers can learn data representations at different levels of abstraction using deep learning (DL) [57]. The two primary issues with traditional 3D reconstruction techniques are as follows. Initially, they require numerous manual designs, which may result in a build-up of errors, but they are barely capable of automatically picking up on the semantic aspects of 3D shapes. Second, they rely heavily on the calibre and content of the images in addition to a properly calibrated camera. By employing deep networks to automatically learn 3D shape semantics from pictures or point clouds, DL-based 3D reconstruction techniques go beyond these obstacles [43,58].

### 4.3. Single-View Reconstruction

Over the years, single-image-based 3D reconstruction has progressed from collecting geometry and texture information from limited types of images to learning neural network parameters to estimate 3D shapes. Real progress in computational efficiency, reconstruction performance, and generalisation capability of 3D reconstruction has been demonstrated. The very first deep-learning-based approaches required real 3D shapes of target objects as supervision, which were extremely difficult to obtain at the time. Some researchers have created images from CAD models to extend datasets; nevertheless, such synthesised data lead to a lack of generalisation and authenticity in the reconstruction results. Some studies have used ground truth 2D and 2.5D projections as supervision and reduced reprojection losses throughout the learning process, such as contour, surface normal, and so on. Later, techniques that compared projections of the reconstructed results with the input to minimise the difference required less supervision. Overall, the field of single-image-based 3D reconstruction is rapidly evolving, and the development of new techniques and architectures is paving the way for more accurate and efficient reconstruction methods. Table 5 provides the list of single-view 3D reconstruction models reviewed in this study.

#### 4.3.1. Point Cloud Representation

**Figure 3:**
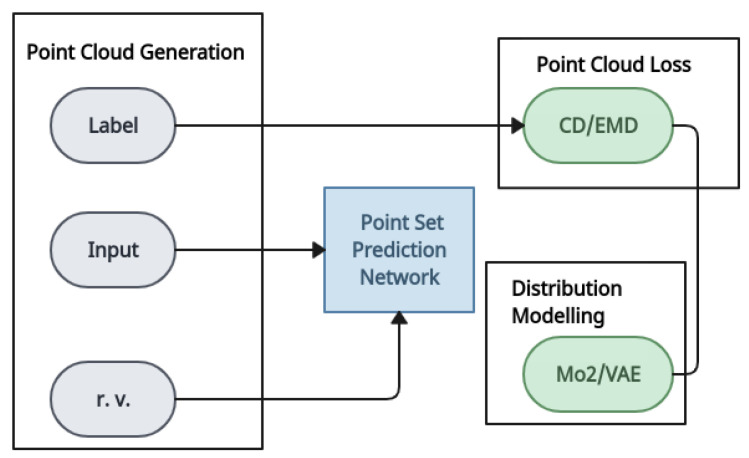
System structure of PointOutNet [9] model.

*PointOutNet* [9]: When compared to voxels, a point cloud is a sparse and memory-saving representation. PointOutNet was proposed to reconstruct objects from a single image in early methods that used point clouds as the output of deep learning networks. PointOutNet has a convolution encoder and two parallel predictor branches. The encoder receives an image as well as a random vector that throws off the prediction. One of the branches is a fully connected branch that captures complex structures, while another is a deconvolution branch that generates point coordinates. This network makes good use of geometric continuity and can produce smooth objects. This research introduced the chamfer distance loss, which is invariant to the permutation of points. This loss function has been adopted by many other models as a regulariser [59,60,61]. The system structure of the PointOutNet model is shown in Figure 3. With the distributional modelling module plugged in, this system may produce several predictions.

**Figure 4:**
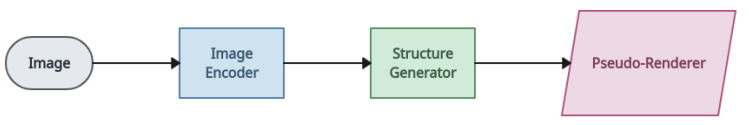
Pipeline of pseudo-renderer [12] model.

*Pseudo-renderer* [12]: The authors of the pseudo-renderer model use 2D convolutional operations to gain improved efficiency. First, they employ a generator to predict 3D buildings at unique view points from a single image. They then employ a pseudo-renderer to generate depth images of corresponding views, which are later used for joint 2D projection optimisation. They predict denser, more accurate point clouds. However, there is usually a limit to the number of points that cloud-based representations can accommodate [62]. When calculating the colour of a pixel, occlusion is taken into consideration by determining a weighted sum of the points’ colours depending on the points’ effects. In order to avoid optimising the occluded points, this model chooses the point that is closest to the camera for a particular pixel [63]. This study uses 2D supervision in addition to 3D supervision to obtain multiple projection images from various viewpoints of the generated 3D shape for optimisation by using a combination of binary cross-entropy loss function with L1 loss function [64]. The pseudo-renderer model’s pipeline is depicted in Figure 4. The authors suggest using a structure generator based on 2D convolutional processes to predict the 3D structure at N perspectives from an encoded latent representation. The 3D structure at each perspective is transformed to the canonical coordinates in order to merge the point clouds. The pseudo-renderer creates depth pictures from fresh perspectives and then uses them to jointly optimise 2D projection. This is based just on 3D geometry and has no learnable parameters.

**Figure 5:**
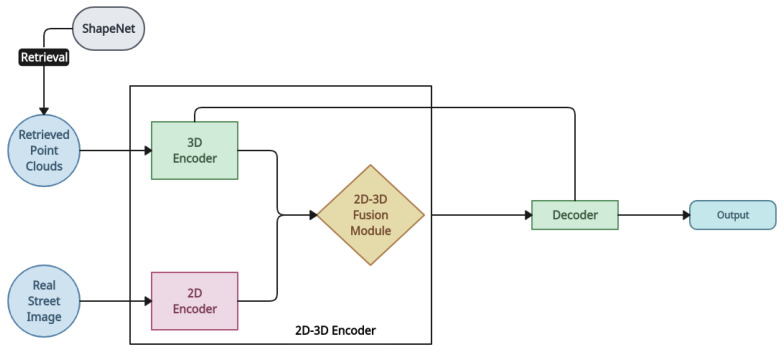
Network architecture of RealPoint3D [13] model.

*RealPoint3D* [13]: The authors of the RealPoint3D model built fine-grained point clouds using a nearby 3D shape as an auxiliary input to the reconstruction network. By giving instructions to the closest form from the ShapeNet, RealPoint3D attempts to recreate 3D models from nature photographs with complicated backgrounds [65,66]. To integrate 2D and 3D features adaptively, the model introduces an attention-based 2D–3D fusion module into the network. By projecting the pixel information from a given 2D image into a 3D space, the method creates point cloud data. It then calculates the chamfer distance and produces a projection loss between the generated and actual point cloud data. The network itself is made up of a 2D–3D fusion module, a decoding section, and an encoding section. The input image’s 2D features and the input point cloud data’s 3D features are extracted throughout the encoding process. The preceding step’s image and spatial characteristics are generated by the 2D–3D fusion module. Finally, the object’s anticipated 3D point clouds are produced by the decoding phase [67]. Figure 5 shows the network architecture of the RealPoint3D model.

**Figure 6:**
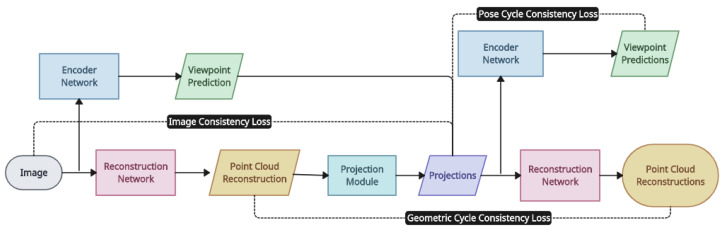
Overview of cycle-consistency-based approach [15].

*A cycle-consistency-based approach* [15]: The authors of this model reconstruct point clouds from images of a certain class, each with appropriate foreground masks. They train the networks in a self-supervised manner using a geometric loss and a pose cycle consistency loss based on an encoder-to-decoder structure, as it is expensive and difficult to collect training data with ground truth 3D annotations. The training impact of multi-view supervision using a single-view dataset is simulated by employing training images with comparable 3D shapes. In addition to two cycle-consistency losses for poses and 3D reconstructions, this model adds a loss-ensuring cross-silhouette consistency [68]. This model uses cycle consistency, which was introduced in CycleGAN [69], to prevent unsupervised learning from annotating 2D and 3D data. It may, however, produce deformed body structures or out-of-view images if unaware of the previous distribution of the 3D features, which would interfere with the training process. Viewed as a basic self-supervised technique, cycle consistency uses the original encoded attribute as the generated image’s 3D annotation [70]. In an analysis-by-synthesis approach, this model uses a differentiable renderer to infer a 3D shape without using ground truth 3D annotation [71]. Figure 6 shows an overview of the cycle-consistency-based approach.

Point-based techniques use less memory, but since they lack connection information, they need extensive postprocessing [72]. Although point clouds are simple 3D representations, they ignore topological relationships [62]. Since point clouds lack a mesh connection structure, further processing is required in order to extract the geometry from the 3D model using this representation [73].

#### 4.3.2. Voxel Representation

**Figure 7:**
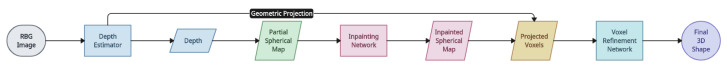
Network architecture of GenRe [20] model.

*GenRe* [20]: A voxel representation is an early 3D representation that lends itself well to convolutional operations. The authors of GenRe train their networks with 3D supervision to predict a depth from a given image in the same view and estimate a single-view spherical map from the depth. They then employ a voxel refinement network to merge two projections and generate a final reconstruction result. This model predicts a 3D voxel grid directly from RGB-D photos using the shape completion approach. This research produces a generalisable and high-quality single-image 3D reconstruction. Others use less supervision in the learning procedure instead of needing 3D ground truth. This model divides the process of converting 2.5D to 3D form into two phases: partial 3D completion and complete 3D completion. This approach differs from the method of directly predicting the 3D shape from 2.5D. To represent the whole surface of the object, the model processes the depth map in turn using an inpainted spherical map and a partial spherical map. Ultimately, the 3D shape is produced by the voxel reconstruction network by combining the back projection of the inpainted spherical image with the depth map. On untrained classes, experimental results demonstrate that the network can also produce outcomes that are more in line with ground truth. These algorithms can rebuild 3D objects with resolutions of up to 128 × 128 × 128 and more detailed reconstruction outcomes. Still, there is a significant difference when it comes to the appearance of actual 3D models [64]. Higher resolutions have been used by this model at the expense of sluggish training or lossy 2D projections, as well as small training batches [74]. Learning-based techniques are usually assessed on new instances from the same category after being trained in a category-specific manner. That said, this approach calls itself category-agnostic [75]. Figure 7 shows the network architecture of the GenRe model.

**Figure 8:**
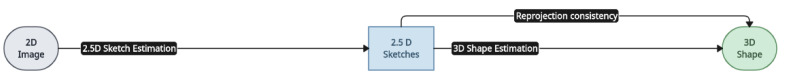
Network architecture of MarrNet [21] model.

*MarrNet* [21]: This model uses depth, normal map, and silhouette as intermediate results to reconstruct 3D voxel shapes and predicts 3D shapes using a reprojection consistency loss. MarrNet contains three key components: (a) 2.5D sketch estimation, (b) 3D shape estimation, and (c) a reprojection consistency loss. From a 2D image, MarrNet initially generates object normal, depth, and silhouette images. The 3D shape is then extrapolated from the generated 2.5D images. It employs an encoding–decoding network in both phases. Finally, a reprojection consistency loss is used to confirm that the estimated 3D shape matches the generated 2.5D sketches. In this work, a multi-view and pose supervised technique is also obtained. This approach avoids modelling item appearance differences within the original image by generating 2.5D drawings from it [76]. Although 3D convolutional neural networks have been used by MarrNet [21] and GenRe [20] to achieve resolutions of up to $128^{3}$, this has only been accomplished with shallow designs and tiny batch sizes, which causes training to go slowly [77]. Due to the global nature of employing image encoders for conditioning, these models exhibit weak generalisation capabilities and are limited by the range of 3D-data-gathering methods employed. Furthermore, in order to guarantee alignment between the predicted form and the input, they need an extra pose estimation phase [78]. This model uses ShapeNet for 3D annotation, which contains objects of basic shapes [79]. Also, it relies on 3D supervision, which is only available for restricted classes or in a synthetic setting [80]. A complete overview is illustrated in Figure 8.

**Figure 9:**
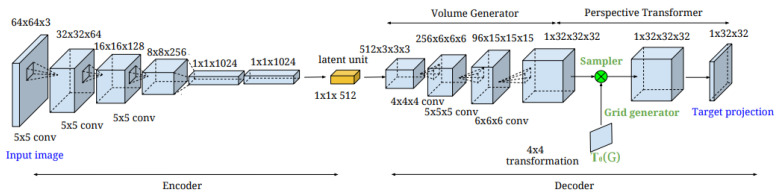
Network architecture of Perspective Transformer Nets [23] model.

*Perspective Transformer Nets* [23]: This method introduces a novel projection loss for learning 2D observations in the absence of 3D ground truths. To reconstruct 3D voxels, the authors employ a 2D convolutional encoder, a 3D up-convolutional decoder, and a perspective transformer network. They reached cutting-edge performance at the time. When rendering a pixel, all of the voxels along a ray that project to that pixel are considered. The final pixel colour can be selected with this model. When displaying voxels, the gradient problem brought on by primitive shape displacement does not arise since a voxel’s location is fixed in three dimensions. Using camera settings, this model projects the voxels from the world space to the screen space and performs more computationally efficient bilinear sampling. Using this strategy, every pixel has an occupancy probability assigned to it. Casting a ray from the pixel, sampling each corresponding voxel, and selecting the one with the highest occupancy probability yields this result [63]. In addition to mainly focusing on inferring depth maps as the scene geometry output, this method has also shown success in learning 3D volumetric representations from 2D observations based on principles of projective geometry [81]. This method requires object masks [82]. Because the underlying 3D scene structure cannot be utilised, this 2D generative model only learns to parameterise the manifold of 2D natural pictures. It struggles to produce images that are consistent across several views [83]. The complete network architecture is illustrated in Figure 9.

**Figure 10:**
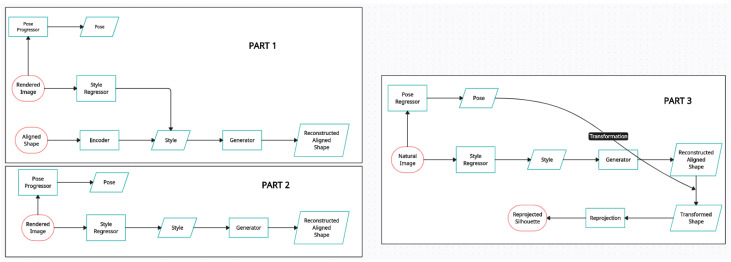
Proposed methods for reconstructing pose-aware 3D voxelised shapes: p-TL (parts 1 and 3) and p-3D-VAE-GAN (parts 2 and 3) [24] models.

*Rethinking reprojection* [24]: The authors of this model, in contrast to the previous research, reconstruct pose-aware 3D shapes from a single natural image. This model uses a well-known, highly accurate, and resilient approach called reprojection error minimisation for shape reconstruction. It demonstrates how well the genuine projection on the image is recreated by an approximated 3D world point [84]. This approach trains shape regressors by comparing projections of ground truths and predicted shapes [85]. Usually, images containing one or a few conspicuous, distinct items are used to test this strategy [86]. The network reconstructs the 3D shape in a canonical posture from the 2D input. The posture parameters are estimated concurrently by a pose regressor and subsequently applied to the rebuilt canonical shape. Decoupling shape and posture lowers the number of free parameters in the network, increasing efficiency [87]. In the absence of 3D labels, this model uses additional 2D reprojection losses to highlight the border voxels for rigid objects [88]. Most of the time, this approach assumes that the scene or object to be registered is either non-deformable or generally static [89]. This representation is limited in terms of resolution [90]. Figure 10 shows the proposed methods of p-TL and p-3D-VAE-GAN models.

**Figure 11:**
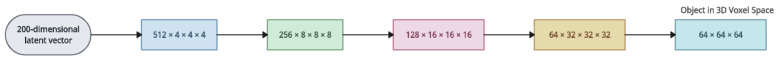
The generator in 3D-GAN [27] model.

*3D-GAN* [27]: The authors of this model present an unsupervised framework that combines adversarial and volumetric convolutional networks to produce voxels from a probabilistic latent space. They enhance the network’s generalisation capacity. Using volumetric convolutions, the developers of this model demonstrated GANs that could create three-dimensional (3D) data samples. They created new items such as vehicles, tables, and chairs. They also demonstrated how to convert two-dimensional (2D) images into three-dimensional (3D) representations of the objects shown in those images [91]. Using this model, visual object networks [92] and PrGANs [93] generate a voxelised 3D shape first, which is then projected into 2D to learn how to synthesise 2D pictures [94]. This approach’s generative component aims to map a latent space to a distribution of intricate 3D shapes. The authors train a voxel-based neural network (GAN) to produce objects. The drawback is that GAN training is notoriously unreliable [95]. Figure 11 shows the generator in the 3D-GAN model mirrored by the discriminator.

Methods to generate voxels frequently do not provide texture or geometric features, and the generating process at high resolution is hampered by the 3D convolution’s large memory footprint and computational complexity [96]. Nevertheless, point cloud and voxel-based models are frequently predictable and only provide a single 3D output [97]. Although point clouds and voxels are more compatible with deep learning architectures, they are not amenable to differentiable rendering or suffer from memory inefficiency problems [98].

#### 4.3.3. Mesh Representation

**Figure 12:**
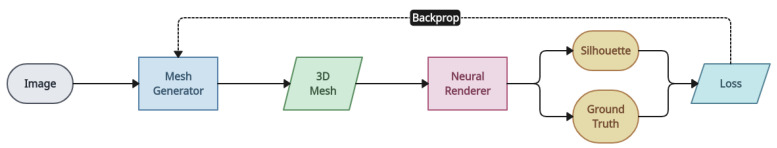
Pipeline for single-image 3D reconstruction [35].

*Neural renderer* [35]: Building differentiable rendering pipelines is the goal of a new discipline called neural rendering, which is making quick strides towards producing controlled, aesthetically realistic rendering [99]. The authors of this model use an integrated mesh rendering network to reconstruct meshes from low-resolution images. They minimise the difference between reconstructed objects and their respective ground truths on 2D silhouettes. The authors suggest a renderer called neural 3D mesh renderer (NMR) and bring up two problems with a differentiable renderer called OpenDR [100]. The gradient computation’s locality is the first problem. Only gradients on border pixels can flow towards vertices due to OpenDR’s local differential filtering; gradients at other pixels are not usable. This characteristic might lead to subpar local minima in optimisation. The derivative’s failure to make use of the target application’s loss gradient—such as picture reconstruction—is the second problem. One technique employed for evaluation involves visualising gradients (without revealing ground truth) and assessing the convergence effectiveness of those gradients throughout the optimisation of the objective function [63]. In the forward pass, NMR carries out conventional rasterisation, and in the backward pass, it computes estimated gradients [101]. For every object instance, the renderings and splits derived from this model offer 24 fixed elevation views with a resolution of 64 × 64 [82]. The objects are trained in canonical pose [72]. This mesh renderer modifies geometry and colour in response to a target image [102]. Figure 12 shows the single-image 3D reconstruction.

**Figure 13:**
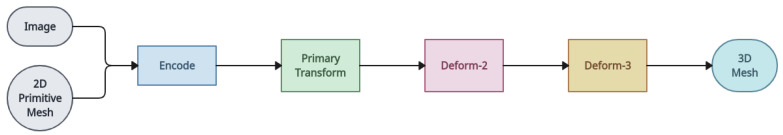
Main network structure of Residual MeshNet [36].

*Residual MeshNet* [36]: To reconstruct 3D meshes from a single image, the authors present this model, a multilayered framework composed of several multilayer perceptron (MLP) blocks. To maintain geometrical coherence, they use a shortcut connection between two blocks. The authors of this model suggest reconstructing 3D meshes using MLPs in a cascaded hierarchical fashion. Three blocks of stacked MLPs are used for hierarchical mesh deformation in the suggested design, along with a ResNet-18 image encoder for feature extraction. To conduct the fundamental shape deformation, the first block, which has one MLP, is supplied with the coordinates of a 2D mesh primitive and image features. The next blocks include many stacked MLPs that concurrently alter the mesh that was previously deformed [103]. The trained model was built on a chamfer distance (CD)-based goal, which promotes consistency between the generated meshes and the ground truth meshes [67]. This work, however, has challenges in reconstructing smooth results with proper triangulation. The majority of mesh learning techniques aim to achieve a desired shape by deforming a template mesh using the learned shape beforehand, since altering the mesh topology is difficult. This model uses progressive deformation and residual prediction, which adds additional details while reducing learning complexity. Despite having no complicated structure, it results in significant patch overlaps and holes [104]. This model is used to produce meshes automatically during the finite element method (FEM) computation process. Although this does not save time, it increases computing productivity [105]. Figure 13 shows the network structure of Residual MeshNet.

**Figure 14:**
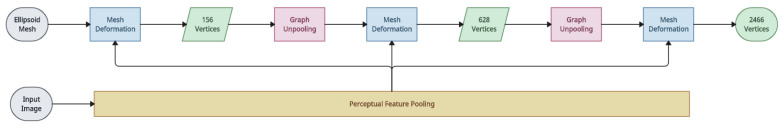
Cascaded mesh deformation network [37].

*Pixel2Mesh* [37]: This model reconstructs 3D meshes of hard objects using a cascaded, graph-based convolutional network to obtain greater realism. The network extracts perceptual features from the input image and gradually deforms an ellipsoid in order to obtain the output geometry. The complete model has three consecutive mesh deformation blocks. Each block enhances mesh resolution and estimates vertex positions, which are later used to extract perceptual image features for the following block. However, several perspectives of the target object or scene must be included in the training data for 3D shape reconstruction, which is seldom the case in real-world scenarios [99]. Figure 14 shows an overview of the Pix2Mesh framework.

Other research, in addition to the above, proposes reconstructing inherent deformations in non-rigid objects. Non-rigid reconstruction tasks from a single image typically require additional information about the target objects, which can be predicted during the process or provided as prior knowledge, such as core structures and parameterised models.

**Figure 15:**
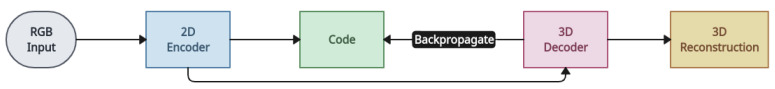
Pipeline of 3D reconstruction using CoReNet [38].

*CoReNet* [38]: This model is a coherent reconstruction network that collaboratively reconstructs numerous objects from a single image for multiple object reconstruction. The authors of this model suggest three enhancements by building on popular encoder–decoder designs for this task: (1) a hybrid 3D volume representation that facilitates the construction of translation equivariant models while encoding fine object details without requiring an excessive memory footprint; (2) ray-traced skip connections that propagate local 2D information to the output 3D volume in a physically correct manner; and (3) a reconstruction loss customised to capture overall object geometry. All objects detected in the input image are represented in a single, consistent 3D coordinate without intersection after passing through a 2D encoder and a 3D decoder. To assure physical accuracy, a ray-traced skip connection is introduced. CoReNet uses a voxel grid with offsets for the reconstruction of scenes with many objects; however, it needs 3D supervision for object placement and identification [82]. Instead of using explicit object recognition, CoReNet used a physical-based ray-traced skip link between the picture and the 3D volume to extract 2D information. Using a single RGB picture, the method reconstructs the shape and semantic class of many objects directly in a 3D volumetric grid [106]. As of late, CoReNet has been able to rebuild many objects on a fixed grid of $128^{3}$ voxels while preserving 3D position data in the global space. Additionally, training on synthetic representations restricts their practicality in real-world situations [107]. Figure 15 shows the pipeline of 3D reconstruction using this model.

Table 6 provides the advantages and limitations of single-view 3D reconstruction models reviewed in this study. In brief, these approaches show the potential of deep learning for 3D object reconstruction using mesh representation. Nevertheless, most of these methods do not have the ability to dynamically change the template mesh’s topology [108]. The majority of these mesh-based techniques do not involve postprocessing, but they frequently call for a deformable template mesh made up of many three-dimensional patches, which results in non-watertight meshes and self-intersections [72].

Numerous organised formats, such as voxel grids, point clouds, and meshes that display heterogeneity per element, are used to store 3D data. For instance, the topology and quantity of vertices and faces might vary throughout meshes. Because of this variability, it is challenging to apply batched operations on 3D data in an effective manner with the tensor-centric primitives offered by common deep learning toolkits such as PyTorch [101].

These studies do not address multi-object analysis, but they do provide intriguing solutions to their particular issues with single object pictures [109]. All that is needed for these tasks is single-view self-supervision. Even with this tremendous advancement, these techniques nonetheless have two main drawbacks: (1) ineffective bottom-up reasoning, in which the model is unable to capture minute geometric features like concavities; and (2) incorrect top-down reasoning, in which the model just explains the input perspective and is unable to precisely recreate the entire 3D object shape [110]. The drawback of this single-category technique is that data cannot be pooled across categories, which might be useful for tasks like viewpoint learning and generalisation to previously unknown categories of objects (zero-shot [111] or few-shot [112] learning) [113]. There are restrictions on the kinds of scenes that can be reconstructed using these methods, as they are designed to only use a single input view at test time [82]. Results from single-view 3D reconstruction are typically incomplete and inaccurate, particularly in cases where there are obstructions or obscured regions [114].

### 4.4. Multiple-View Reconstruction

The apparent uncertainty in the object is decreased and the number of occluded portions is increased when images taken from different angles are fed into the network. Traditionally, there have been two kinds of reconstruction from several perspectives. Rebuilding a static item from a number of images is the first step; reconstructing a moving object’s three-dimensional structure from a movie or several frames is the second. In order to match up the incomplete 3D shapes into a full one, both of these algorithms use images to estimate the camera posture and matching shape. As a result, three-dimensional alignment and posture estimation are challenging. First, deep learning techniques are introduced into multi-image reconstruction to address this problem. Next, from the input images, 3D shapes are immediately generated by deep neural networks. Moreover, the rebuilding procedure takes a lot less time when end-to-end structures are used. Table 7 provides the list of multi-view 3D reconstruction models reviewed in this study.

#### 4.4.1. Point Cloud Representation

*3D34D* [17]: The authors of this model employ a UNet encoder, producing feature maps to produce geometry-aware point representations of object categories unseen during training. For 3D object reconstruction, this study employs multi-view images with ground truth camera postures and pixel-aligned feature representations. A stand-alone 3D reconstruction module that was trained using ground truth camera postures is used by this model [115]. This work has made generalisation a clear goal. The goal of this study is to obtain a more expressive intermediate shape representation by locally assigning features and 3D points [116]. This is an object-centred approach. This work was the first to examine the generalisation characteristics of shape reconstruction using previously unknown shape categories. This approach emphasises reconstruction from many perspectives, uses continuous occupancies, and evaluates generalisation to previously undiscovered categories [117]. The study focused on reconstruction from several perspectives and examined feature description bias for generalisation [118]. While this 3D reconstruction technique performs admirably on synthetic objects rendered with a clear background, it may not translate well to actual photos, novel categories, or more intricate object geometries [75]. According to this research, contemporary learning-based computer vision techniques are unable to generalise to data that is not distributed evenly [119].

**Figure 16:**
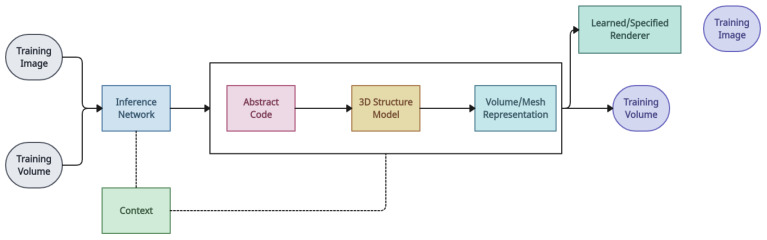
Proposed framework of unsupervised learning of 3D structure from images [18].

*Unsupervised learning of 3D structure from images* [18]: The authors of this model train deep generative models of 3D objects in an end-to-end fashion and directly from 2D images without the use of 3D ground truth, and then reconstruct objects from 2D images via probabilistic inference. This purely unsupervised method is built on sequential generative models and can generate high-quality samples that represent the multi-modality of the data. With a primary focus on inferring depth maps as the scene geometry output, this study has demonstrated success in learning 3D volumetric representations from 2D observations using the concepts of projective geometry [81]. In [120], synthesised data are used. Ref. [121] explores the use of 3D representations as inductive bias in generative models. Using adversarial loss, the technique presented in [122] usually optimises 3D representations to provide realistic 2D images from all randomly sampled views. An effort based on policy gradient algorithms performs single-view 3D object reconstruction using the non-differentiable OpenGL renderer with this model. Nevertheless, only basic and coarse forms may be recreated in the collection [63]. Figure 16 shows the overall framework for this model.

Overall, these techniques offer significant progress in the area of multi-view reconstruction, enabling the generation of 3D models from 2D data in a more accurate and efficient manner. There is still room for improvement, especially when it comes to better alignment accuracy and estimating camera poses. Further research and development in this area could lead to even more sophisticated techniques for generating 3D models from multiple images.

#### 4.4.2. Voxel Representation

**Figure 17:**
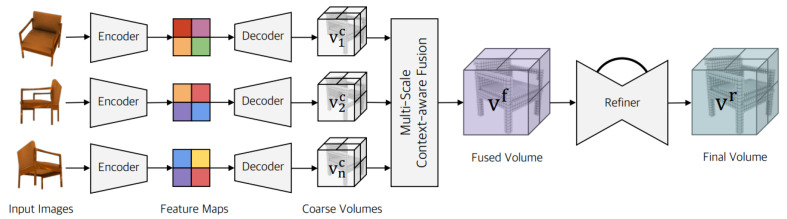
Proposed framework of Pix2Vox++ network [30].

*Pix2Vox++* [30]: The authors of this model listed three limitations for RNN-based methods. First, permutation variance prevents RNNs from reliably estimating the 3D geometry of an item when they are presented with the same collection of pictures in various sequences. Second, the input pictures cannot be properly used to improve reconstruction outcomes due to RNNs’ long-term memory loss. Finally, as input pictures are analysed sequentially without parallelisation, RNN-based algorithms take a long time. To overcome these limitations, the authors proposed an encoder–decoder structure framework called Pix2Vox [123] based on RNNs. The authors introduced Pix2Vox++ [30] by making some improvements to the previously created Pix2Vox [123] model. In the Pix2Vox++ [30] network, the authors replaced the backbone of Pix2Vox [123], VGG, with ResNet. The authors of this model proposed Pix2Vox++ to generate a coarse volume for each input image. They fuse all of the coarse volumes using a multi-scale context-aware fusion module, followed by a refiner module to correct the fused volume. Primarily using synthetic data, such as from ShapeNet, this model learns to rebuild the volumetric representation of basic objects [124]. Pix2Vox++’s reconstruction findings are able to precisely recreate the general shape but are unable to provide fine-grained geometries [125]. Because of memory limitations, the model’s cubic complexity in space results in coarse discretisations [126]. The visual information is transferred from the image encoder to the 3D decoder using only the feature channels (such as element-wise add, feature concatenation, and attention mechanism). The 3D decoder only receives implicit geometric information with limited semantic attributes, which serves as guidance for shape reconstruction. The decoder can quickly detect and recover such geometric information. On the contrary, the particular, detailed shape of these attributes will be determined by the detailed semantic attributes. However, throughout the reconstruction process, the decoder will seldom discover these semantic properties since they are intricately intertwined with one another in image features. The resolution for voxel data is often constrained due to the cubic growth of the input voxel data, and further raising the resolution would result in unacceptably high computing costs [127]. The accuracy of the method will become saturated when the number of input views exceeds a specific scale (e.g., 4), indicating the challenge of acquiring complementary information from a large number of independent CNN feature extraction units [128]. Figure 17 shows the proposed framework for this model.

**Figure 18:**
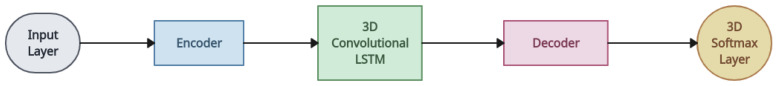
An overview of the 3D-R2N2 network [11].

*3D-R2N2* [11]: Deeply influenced by the conventional LSTM framework, 3D-R2N2 generates 3D objects in occupancy grids with only bounding box supervision. In an encoder–LSTM–decoder structure, it merges single- and multi-view reconstruction. The 3D convolutional LSTM selectively updates hidden representations via input and forget gates. It successfully manages self-occlusion and refines the reconstruction result progressively as additional observations are collected. An overview of the network is presented in Figure 18. Despite the ability to preserve earlier observations, methods based on such structures may fail when presented with similar inputs and are restricted in their ability to retain features in early inputs. Using encoder–decoder architectures, this technique converts RGB image partial inputs into a latent vector, which is then used to predict the complete volumetric shape using previously learned priors. Fine shape features are lost in voxel-based methods, and since their normals are not smooth when produced, voxels look very different from high-fidelity shapes [95]. This CNN-based method only works with coarse 64 × 64 × 64 grids [129]. This approach has significant memory use and computational overhead [61]. Since voxels are logical extensions of image pixels, cutting-edge methods for shape processing may be transferred from image processing. Nevertheless, low-resolution outcomes are typically produced because voxel representations are limited by GPU memory capacity [130].

*Weak recon* [31]: This method explores an alternative to costly 3D CAD annotation and proposes using lower-cost 2D supervision. Through a ray-trace pooling layer that permits perspective projection and backpropagation, the proposed method leverages foreground masks as weak supervision. By constraining the reconstruction to remain in the space of unlabelled real 3D shapes, this technique makes use of foreground masks for 3D reconstruction. Using ray-tracing pooling, this model learns shapes from multi-view silhouettes and applies a GAN to further limit the ill-posed issue [131]. This method is limited to low-resolution voxel grids [132]. The authors decided to employ GANs to represent 2D projections rather than 3D shapes when investigating adversarial nets for single-image 3D reconstruction. However, their reconstructions are hampered by this weakly supervised environment [133].

**Figure 19:**
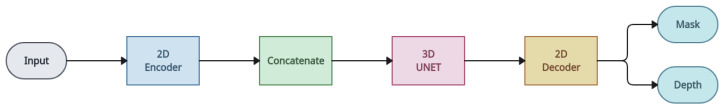
An overview of the shape-learning approach [32].

*Relative viewpoint estimation* [32]: The authors of this model propose teaching two networks to address alignment without 3D supervision: one to estimate the 3D shape of an object from two images of different viewpoints with corresponding pose vectors and predict the object’s appearance from a third view; and the other to evaluate the misalignment of the two views. They predict a transformation that optimally matches the bottleneck features of two input images during testing. Their networks are also focused on generalising previously unseen objects. When estimating relative 3D poses among a group of little or non-overlapping RGB(-D) images, perspective variation is significantly more dramatic in regions where few co-visible regions are identified, making matching-based algorithms inappropriate. The authors of this model suggest using the hallucination-then-match paradigm to overcome this difficulty [134]. The authors point out that supplying an implicit canonical frame by using a reference image and formulating posture estimation as predicting the relative perspective from this view are the basic requirements to make zero-shot pose estimation a well-posed issue. Unfortunately, this technique does not extend to the category level; it can only predict posture for instances of a single item [135]. Figure 19 shows an overview of the shape-learning approach of this model.

Table 8 provides the advantages and limitations of multi-view 3D reconstruction models reviewed in this study. Point clouds, voxel grids, and mesh scene representations, on the other hand, are discrete, restricting the amount of spatial resolution that can be achieved, meaning they only sample the smooth surfaces underneath a scene sparingly, and they frequently require explicit 3D supervision [83].

## 5. Registration

Determining the correlation between point cloud data of the same image acquired from several methods might be useful in some scenarios. By calculating the transformation for the optimal rotation and translation across the point cloud sets, 3D point cloud registration algorithms reliably align different overlapping 3D point cloud data views into a full model (in a rigid sense). The distance in a suitable metric space between the overlapping regions of two distinct point cloud sets is small in an ideal solution. This is difficult since noise, outliers, and non-rigid spatial transformations all interfere with the process. Finding the optimal solution becomes significantly more difficult when there is no information about the starting posture of various point cloud sets in space or the places where the sets overlap. Table 9 provides the list of 3D registration models reviewed in this study.

### 5.1. Traditional Methods

Traditional 3D registration methods can be classified based on whether the underlying optimisation method used is global or local. The most well-known works in the global category are based on global stochastic optimisation using genetic algorithms or evolutionary algorithms. However, their main drawback is the computation time. On the other hand, the majority of studies performed in 3D registration nevertheless have local optimisation methods.

*CPD* [136]: The Coherent Point Drift (CPD) algorithm considers the alignment as a probability density estimation problem where one point cloud set represents the Gaussian mixture model centroids and the other represents the data points. The transformation is estimated by maximising the probability of fitting the centroids to the second set of points. The movement is forced to move coherently as a group to preserve the topological structure. The authors introduced this approach, which uses the methodology for maximum likelihood parameter estimation and establishes a probabilistic framework based on Gaussian mixture models (GMMs) [147]. Registration was reformulated by the authors as a probability density estimation issue. The first set of points served as the centroids of the GMMs that were fitted using likelihood maximisation to the data or points from the second set. To ensure that the centroids moved coherently, extra effort was taken [148]. While GMM-based methods might increase resilience against outliers and bad initialisations, local search remains the foundation of optimisation [149].

*PSR-SDP* [137]: The authors of this model studied the registration of point cloud sets in a global coordinate system. In other words, with the original set of *n* points, we want to find the correspondences between (subsets of) the original set and *m* local coordinate systems, respectively. The authors consider the problem as a semi-definite program (SDP) within the application of Lagrangian duality, and this allows for verifying the global optimality of a local minimiser in a significantly faster manner. The registration of numerous point sets is solved by this approach using semi-definite relaxation. By using a convex SDP relaxation, the non-convex constraint is relaxed [150]. Lagrangian duality and SDP relaxations were used to tackle the multiple point cloud registration problem. This problem was investigated further in this model, where it was demonstrated that the SDP relaxation is always tight under low-noise regimes [151]. A study of global optimality requirements for point set registration (PSR) with incomplete data was presented using this approach. This approach used Lagrangian duality to provide a primal problem candidate solution, allowing it to retrieve the associated dual variable in closed form. This approach provides poor estimates even in the presence of a single outlier because it assumes that all measurements are inliers (i.e., have little noise), a situation that rarely occurs in practice [152].

**Figure 20:**
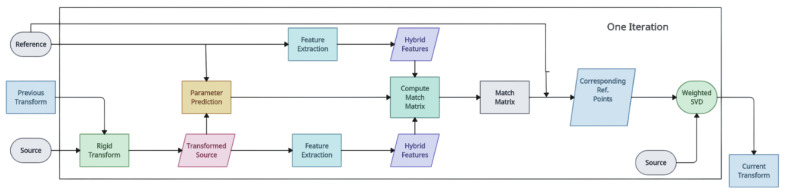
An overview of the RPM-Net network [139].

*RPM-Net* [139]: RPM-Net inherits the idea of the RPM algorithm, introduces deep learning to desensitise the initialisation, and improves network convergence with learned fusion features. In this method, the initialisation assignments are based on the fusion of hybrid features from a network instead of spatial distances between points. The optimal annealing parameters are predicted by a secondary network, and a modified chamfer distance is introduced to evaluate the quality of registration. This method outperforms previous methods and handles missing keypoints and point cloud sets with partial visibility. RPM-Net presents a deep-learning-based method for rigid point cloud registration that is more resilient and less susceptible to initialisation. The network created by this approach is able to solve the partial visibility of the point cloud and obtain a soft assignment of point correspondences [150]. This model’s feature extraction is geared particularly towards artificial, object-centric point clouds [153]. By leveraging soft correspondences that are calculated from the local feature similarity scores to estimate alignment, this approach avoids the non-differentiable nearest-neighbour matching and RANSAC processes. RPM-Net also makes use of surface normal data [154]. Because of matches that are heavily tainted by outliers, this model’s resilience and applicability in complicated scenarios does not always live up to expectations [155]. This approach looks for deep features to find correspondences; however, the features that are taken out of point clouds have a low capacity to discriminate, which results in a high percentage of false correspondences and severely reduces the accuracy of registration. In order to establish soft correspondences from local characteristics, which might boost resilience but reduce registration accuracy, RPM-Net suggests a network that predicts the ideal annealing parameters [156]. Figure 20 shows the network architecture of this model.

### 5.2. Learning-Based Methods

**Figure 21:**
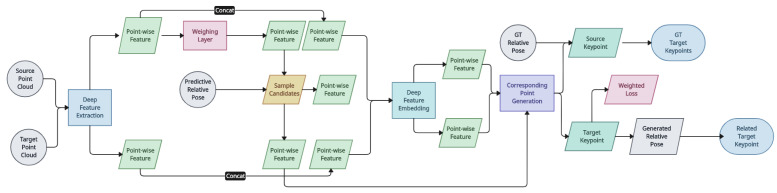
The architecture of DeepICP [140].

*DeepICP* [140]: This is an early end-to-end framework achieving comparable registration accuracy to the state-of-the-art traditional methods for point cloud registration. The algorithm utilises PointNet++ [157] to extract local features, followed by a point-weighting layer that helps select a set of keypoints. Once a set of candidate keypoints is selected from the target point cloud set, they pass through a deep-feature-embedding operation together with the keypoints of the source set. Finally, a corresponding point generation layer takes the embeddings and generates the final result. Two losses are incurred: (1) the Euclidean distance between the estimated corresponding points and the ground truth under the ground truth transformation, and (2) the distance between the target under the estimated transformation and the ground truth. These losses are combined to consider both global geometric information and local similarity. By creating a connection using the point cloud’s learned attributes, this study improved the conventional ICP algorithm using the neural network technique. This method takes a large amount of training time on the dataset, despite its good performance. If the test data change significantly from the training data, the algorithm’s output will not be optimal. Consequently, there are stringent data limits with the neural-network-based enhanced ICP technique [158]. A solution to the point cloud registration problem has been offered [159]. Rather than utilising ICP techniques, this approach might directly match the local and target point clouds in addition to extracting descriptors via neural networks [160]. It still takes a lot of computing effort to combine deep learning with ICP directly [150]. The architecture of the proposed end-to-end learning network for 3D point cloud registration is demonstrated in Figure 21.

*3DSmoothNet* [143]: 3DSmoothNet matches two point cloud sets with a compactly learned 3D point cloud descriptor. At first, the model computes the local reference frame of the area near the randomly sampled keypoints. This is followed by the near areas being transformed into voxelised smoothed density value representations [161]. Then, the local feature of each keypoint is generated by 3DSmoothNet. The features extracted by this cloud descriptor will be utilised by a RANSAC approach for producing registration results. The proposed 3D point cloud descriptor outperforms traditional binary-occupancy grids, and it is the first learned, universal matching method that allows transferring trained models between modalities. For feature learning, this approach suggests a rotation-invariant handcrafted feature that is fed into a deep neural network. Deep learning is used as a feature extraction technique in all these strategies. Their goal is to estimate robust correspondences by learning distinguishing characteristics through the development of complex network topologies or loss functions. This experiment demonstrates that while applying deep learning directly will not ensure correctness, applying mathematical theories of registration directly will require enormous amounts of computing effort [150]. This approach is designed to mitigate voxelisation and noise artefacts. The receptive field is limited to a predetermined size, and the computational cost is significantly increased by this early work’s outstanding performance, which is still based on individual local patches [153]. Fully convolutional geometric features (FCGFs) is the fastest feature extraction method and is 290 times faster than 3DSmoothNet [162].

**Figure 22:**
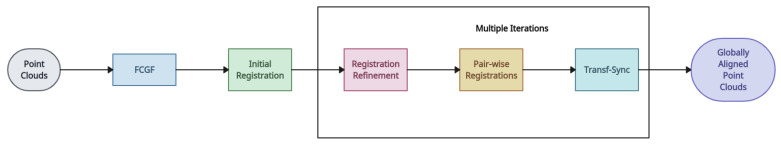
Proposed pipeline for 3D multi-view registration [145].

*3D multi-view registration* [145]: Following 3DSmoothNet, the authors proposed a method that formulates conventional two-stage approaches (typically an initial pairwise alignment followed by a global refinement) in an end-to-end learnable convention by directly learning and registering all views in a globally consistent fashion. Their work improves a point cloud descriptor studied in [162], using a soft correspondence layer that pairs different sets to compute primary matches. These matches are then fed to a pairwise registration block to obtain transformation parameters and corresponding weights. Finally, these weights and parameters are globally refined by a novel iterative transformation synchronisation layer. This work is the first end-to-end algorithm for joint learning of both stages of the registration problem. This model outperforms previous two-stage algorithms with higher accuracy and less computational complexity. This method utilises FCGF [162] to solve the multi-way registration problem [163]. The primary use for this technique is indoor point clouds [164]. Figure 22 shows the proposed pipeline for this method.

Table 10 provides the advantages and limitations of 3D registration models reviewed in this study. This category offers the following two benefits: (1) A point feature based on deep learning may offer reliable and precise correspondence searches. (2) By applying a straightforward RANSAC approach, the correct correspondences might result in accurate registration outcomes. Nevertheless, there are limitations to these kinds of methods: (1) A lot of training data are required. (2) If there is a significant distribution discrepancy between the unknown scenes and the training data, the registration performance in such scenes drastically decreases. (3) To learn a stand-alone feature extraction network, they employ a different training procedure. In addition to registration, the learned feature network is used to determine point-to-point matching [150].

## 6. Augmentation

The proliferation of 3D data collection equipment and the rising availability of 3D point cloud data are the result of recent advancements in 3D sensing technology. Despite the fact that 3D point clouds offer extensive information on the entire geometry of 3D objects, they are frequently severely flawed by outliers, noise, and missing points. Many strategies, including outlier removal, point cloud completion, and noise reduction, have been proposed to solve these problems; however, the implementation and application differ. While point cloud completion techniques try to fill in the missing portions of the point cloud to provide a comprehensive representation of the object, outlier removal strategies try to detect and eliminate points that do not adhere to the overall shape of the object. On the other hand, noise suppression approaches work to lessen the impact of random noise in the data in order to enhance the point cloud’s quality and accuracy. Table 11 provides the list of 3D augmentation models reviewed in this study.

### 6.1. Denoising

While better data gathering methods may result in higher-quality data, noise in point clouds is unavoidable in some circumstances, such as outdoor scenes. A number of denoising methods have been put forward to stop noise from affecting point cloud encoding. Local surface fitting (e.g., jets or MLS surfaces), local or non-local averaging, and statistical presumptions on the underlying noise model are examples of early conventional approaches. Since then, learning-based techniques have been put forward that, in the majority of situations, perform better than traditional solutions.

**Figure 23:**
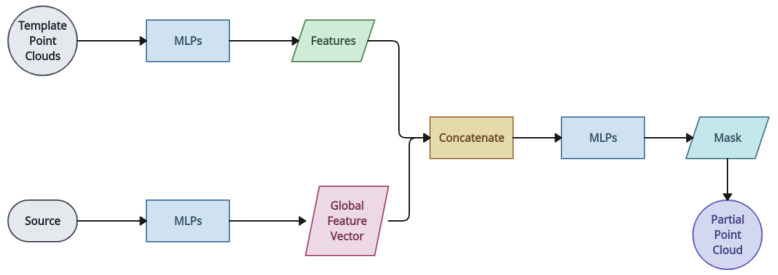
Architecture of MaskNet [165].

*MaskNet* [165]: The authors of this model presented MaskNet for determining outlier points in point clouds by computing a mask. The method can be used to reject noise in even partial clouds in a rather computationally inexpensive manner. This approach, which uses learning-based techniques to estimate global descriptors of each point in the point cloud in addition to a global feature of the point cloud, was presented to address the sparse overlap of point clouds. After that, a predicted inlier mask is used to compute the transformation using these features. This model’s ability to effectively tackle the partial-to-partial registration problem is one of its key advantages. However, this model’s primary drawback is that it requires the input of both a partial and complete point cloud [172]. It requires a point cloud without outliers as a template. Voxelisation or projection are required to convert the initial point clouds into structured data because of the chaos of point clouds. Due to the inevitable rise in computing load and loss in geographical information in certain categories, this process results in issues with significant time consumption and inaccuracy [173]. The feature interaction module of MaskNet is meant to take two point clouds as input and output the posterior probability [174]. To anticipate whether points in template point clouds coincide with those in source point clouds, it makes use of a PointNet-like network. But only in the template point cloud can it identify the overlapping points [175]. One typical issue with raw-point-based algorithms is that they assume a considerable overlap or good starting connections between the provided pair of point sets [176]. MaskNet is not easily transferred to other tasks or real-world situations due to its high sensitivity to noise [177]. According to this method, the extracted overlapping points are assumed to be entirely correct, and they are thought to have equivalent points. However, the accuracy of the overlapping spots that the network estimates cannot be guaranteed [178]. Figure 23 shows the architecture of this model.

However, all of the aforesaid deep learning approaches are fully supervised and require pairs of clean and noisy point clouds.

*GPDNet* [166]: The authors of this model proposed a new graph convolutional neural network targeted at point cloud denoising. The algorithm deals with the permutation-invariance problem and builds hierarchies of local or non-local features to effectively address the denoising problem. This method is robust to high levels of noise and also has structured noise distributions. In order to regularise the underlying noise in the input point cloud, GPDNet suggests creating hierarchies of local and non-local features [179]. Edge-conditioned convolution (ECC) [180] was further expanded to 3D denoising problems using this approach [181]. The two primary artefacts that affect this class of algorithms are shrinkage and outliers, which result from either an overestimation or an underestimation of the displacement [182]. The point clouds’ geometric characteristics are often oversmoothed using GPDNet [183].

**Figure 24:**
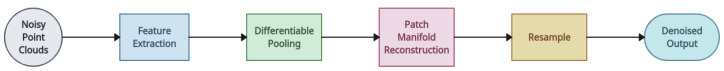
Illustration of the proposed DMR network [167].

*DMR* [167]: The authors of this model presented a novel method to use differentiably subsampled points for learning the underlying manifold of a noisy point cloud. The proposed algorithm is different from the aforementioned methods as it resembles more of a human-like cleaning of a noisy point cloud using multi-scale geometric feature information as well as supervision from ground truths. This network can also be trained in an unsupervised manner. A simple implementation of the graph convolutional network (GCN) is unstable as the denoising process mostly deals with local representations of point neighbourhoods. In order to learn the underlying manifold of the noisy input from differentiably subsampled points and their local features with minimal disruption, DMR relies on dynamic graph CNN (DGCNN) [184] to handle this problem [179]. In this model, the patch manifold reconstruction (PMR) upsampling technique is straightforward and efficient [185]. This method’s downsampling step invariably results in detail loss, especially at low noise levels, and it could also oversmooth by removing some useful information [182]. The goal of these techniques is to automatically and directly learn latent representations for denoising from the noisy point cloud. Its overall performance on noise in the actual world is still restricted though [186]. Figure 24 shows the architecture of this model.

### 6.2. Upsampling

In 3D point cloud processing, upsampling is a typical challenge when the objective is to produce a denser set of points that faithfully depicts the underlying geometry. Though the uneven structure and lack of spatial order of point clouds present extra obstacles, the problem is analogous to the image super-resolution problem. Points had to be adjusted in the early, traditional point cloud upsampling techniques, which were optimisation-based. Although these approaches frequently yielded satisfactory results, their application was limited since they assumed smooth underlying geometry. Recently, data-driven approaches have emerged for point cloud upsampling, which have demonstrated significant improvements over traditional methods.

**Figure 25:**
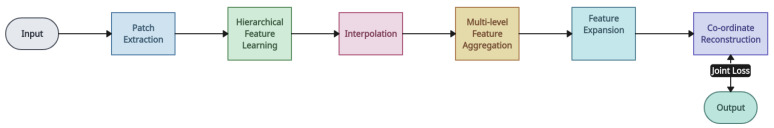
Architecture of PU-Net [168].

*PU-Net* [168]: PU-Net is one such approach that uses a multi-branch convolutional unit to expand the set of points in a point cloud by learning multi-level features for each point. During the end-to-end training of PU-Net, both reconstruction loss and repulsion loss are jointly utilised to improve the quality of the output. PU-Net learns the representation from the raw point dataset using unsupervised methods. This model learns sparse and irregular point clouds. Each point’s multi-level features are learned, and the enlarged feature is obtained by applying multi-branch convolution. This feature is then divided to rebuild the point cloud. PU-Net consists of four components: patch extraction, which gathers point clouds of different sizes; point feature embedding, which extracts the point clouds’ local and global geometric information; feature expansion, which increases the number of features; and coordinate reconstruction, which implements the expanded features’ 3D coordinates [187]. It is a LiDAR-based technique that uses raw LiDAR scans to learn high level point-wise information. From each high-dimensional feature vector, many upsampled point clouds are then reconstructed [188]. Recovering the 3D shape of objects that have only partially been observed can be achieved to a limited extent by upsampling point clouds. Moreover, there would be a noticeable increase in latency if the whole point cloud was upsampled. A low-density point cloud can be converted to a high-density one via point cloud upsampling. Nevertheless, during training, they require high-density point cloud ground truths [189]. This model only learns spatial relationships at a single level of multi-step point cloud decoding via self-attention [190]. With exceptionally sparse and non-uniform low-quality inputs, this network might not generate findings that are believable. PU-Net replicates the point features and processes each copy independently using different MLPs in order to upsample a point collection. But the enhanced features would be too close to the inputs, degrading the quality of the upsampling [191]. The detailed architecture of PU-Net is presented in Figure 25.

**Figure 26:**
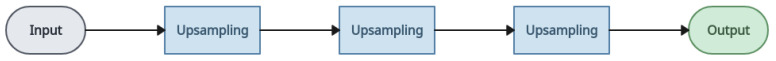
Overview of MPU with 3 levels of detail [169].

*MPU* [169]: The authors of this model proposed an adaptive patch-based point cloud upsampling network that was inspired by recent neural image super-resolution methods. This network is trained end-to-end on high-resolution point clouds and emphasises a certain level of detail by altering the spatial span of the receptive field in various steps. It is a LiDAR-based technique that uses raw LiDAR scans to learn high-level point-wise information. From each high-dimensional feature vector, it then reconstructs many upsampled point clouds [188]. Recovering the 3D shape of objects that have only partially been observed can be achieved to a limited extent by upsampling point clouds. Moreover, there would be a noticeable increase in latency if the whole point cloud was upsampled. A low-density point cloud can be converted to a high-density one via point cloud upsampling. Nevertheless, during training, they require high-density point cloud ground truths [189]. This model only learns spatial relationships at a single level of multi-step point cloud decoding via self-attention [190]. With exceptionally sparse and non-uniform low-quality inputs, this network might not generate findings that are believable. A multi-step progressive upsampling (MPU) network was provided by the authors in order to reduce noise and preserve information. This approach divides a 16× upsampling network into four consecutive 2× upsampling subnets to upsample a point set incrementally in numerous phases. The training procedure is intricate and needs more subgroups for a greater upsampling rate, even if details are better maintained in the upsampled output [191]. The amount of computing memory used during training is higher. More significantly, this approach cannot be used for completion tasks and is restricted to upsampling sparse locations [192]. Figure 26 shows an overview of the MPU network with three levels of detail.

Even with the recent advancements, point cloud upsampling still faces difficulties, particularly when managing intricate structures with a range of densities and imperfections. Another problem is that the quality of the input has a significant impact on the quality of the point clouds that are created. More investigation is required to create point cloud upsampling algorithms that are more effective and efficient in order to overcome these obstacles.

### 6.3. Downsampling

In practical settings, the point cloud often contains a large number of points due to the use of high-density data acquisition sensors. While some applications benefit from this density, increased computation and low efficiency are common issues. One conventional approach is downsampling the point cloud using a neural network.

**Figure 27:**
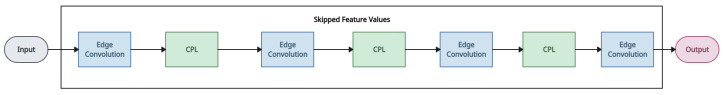
General overview of CP-Net [170].

*CP-Net* [170]: The authors of this model propose a critical points layer (CPL) that downsamples the points adaptively based on learned features. The following network layer receives the points with the most active features from this critical points layer [193]. CPL globally filters out unimportant points while preserving the important ones. The proposed CPL layer can be used with a graph-based point cloud convolution layer to form CP-Net. When using this approach, the final representations typically retain crucial points that take up a significant amount of channels [194]. This graph-based downsampling approach uses K-nearest neighbours (K-NNs) to locate neighbouring points, in contrast to the majority of graph-based point cloud downsampling techniques. In addition, the global downsampling technique known as the critical points layer (CPL) has very high computing efficiency. A graph-based layer and the suggested layer can be used to create a convolutional neural network [195]. It is not possible to describe the underlying geometric structures of points or to properly capture the non-local dispersed contextual correlations in geographical locations and semantic information using this point-based approach that has recently been presented, which needs complex network designs to aggregate local features [196,197], as shown in Figure 27.

**Figure 28:**
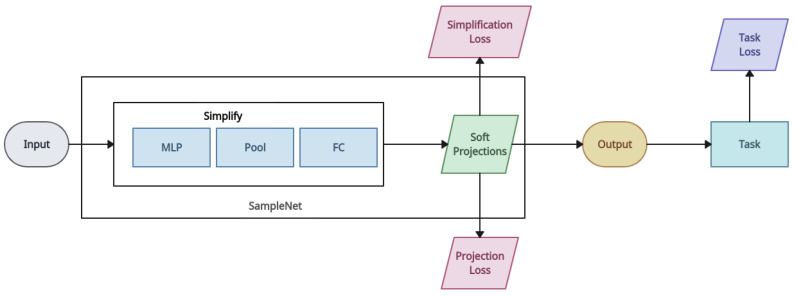
Training of the proposed sampling method [171].

*SampleNet* [171]: SampleNet is a differentiable sampling network used for reconstruction and classification tasks in point clouds [198]. It introduces a differentiable relaxation for point cloud sampling by approximating sampled points as a mixture of points in the original point cloud. This network can be used as a front to networks for multiple tasks, unlike conventional approaches that do not consider the downstream task. With this model, the sampling procedure for the representative point cloud classification problem becomes differentiable, allowing for end-to-end optimisation [194]. For the downstream tasks, SampleNet suggests a learned sampling strategy [199]. In this work, the creation of additional data points is how sampling is accomplished [200]. This neural network is intended to choose the keypoints more accurately [201]. By choosing already-existing points from the point cloud, this method restricts itself [202]. The model fails to attain a satisfactory equilibrium between maintaining geometric features and uniform density. Following the sampling process, the original point clouds’ moving least squares (MLS) surfaces are modified [203]. There are two major drawbacks to this method: it requires supervised annotations in the form of labels. The first is the restricted scalability due to the high cost of building a systematic annotation strategy and obtaining human annotations. Second, when several objects are present in labelled data obtained in the field (e.g., from a vehicle-mounted LiDAR sensor), it becomes very difficult and time-consuming to determine whether points in a point cloud of 10,000 points belong to a car, the street, or another automobile [204]. Figure 28 shows the training of the proposed method.

Table 12 provides the advantages and limitations of the 3D augmentation models reviewed in this study. All things considered, traditional techniques for downsampling point clouds frequently result in more computation or the removal of significant points. SampleNet [171] and CP-Net [170] provide answers to these problems. While SampleNet [171] presents a differentiable relaxation for point cloud sampling, which may be used as a front to networks for numerous tasks, CP-Net [170] globally filters away unnecessary points while maintaining the significant ones. These recent developments provide the groundwork for future improvements in downstream tasks and are critical to 3D point cloud processing.

## 7. Point Cloud Completion

Point clouds are the most widely used depiction of 3D data, and they are frequently used in practical applications. However, acquired point clouds are typically highly incomplete and sparse due to self-occlusion and poor sensor resolution, which hinders further applications. Thus, recovering complete point clouds is an essential task, the main goals of which are to densify sparse surfaces, infer missing sections, and preserve the details of incomplete observations. Because point clouds are inherently chaotic and unstructured (especially when taken from real-world settings), their completion is typically non-trivial. Table 13 provides the advantages and limitations of point cloud completion models reviewed in this study.

**Figure 29:**
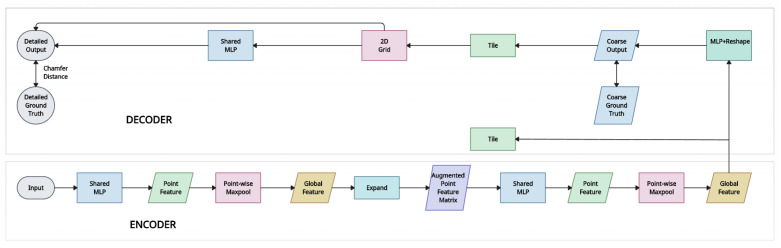
Architecture of PCN [205].

*PCN* [205]: A coarse-to-fine point set generator and a permutation-invariant, non-convolutional feature extractor were combined by the model’s designers to create a single, end-to-end trained network. PCN is an encoder–decoder network, where the encoder produces a k-dimensional feature vector from the input point cloud. Using this feature vector as input, the decoder generates a coarse and detailed output point cloud. The loss function, which is used to train the entire network via backpropagation, is calculated between the outputs of the decoder and the ground truth point cloud. The authors did not specifically mandate the network to maintain the input points in its output, in contrast to an autoencoder. On the contrary, the network acquires knowledge of a projection from the space of incomplete observations to the space of fully formed shapes. This network’s primary drawback is that the encoder requires training data to be prepared in partial shapes since it expects a test input that is identical to the training data [95]. The lack of utilisation of object completion and shape creation architectures, like PCN [205], by 3D object detectors during the LiDAR point cloud inference could result in improved detection performance [206]. This point cloud completion method’s max-pooling process during the encoding phase, where fine-grained information is lost and scarcely recoverable during the decoding phase, is its bottleneck [61]. This model, which focuses on object-level completion, works under the assumption that a single item has been found manually and that the input consists only of the points on this object. Consequently, this model is not appropriate for the goal of object detection [189]. Figure 29 shows the architecture of PCN.

*Unpaired scan completion network* [207]: The authors provide an unpaired point-based scan completion technique that can be learned without having explicit correspondence between example complete shape models (like synthetic models) and incomplete point sets (like raw scans). Due to the lack of specific instances of real complete scans required by this network, large-scale real 3D scans that are already available (unpaired) can be used directly as training data. This is accomplished by creating a generative adversarial network (GAN), in which the input is transformed into a suitable latent representation by a generator, also known as an adaptation network, so that a discriminator is unable to distinguish between the transformed latent variables and the latent variables derived from training data (i.e., whole-shape models). Working in two distinct latent spaces with independently learned manifolds of scanned and synthesised object data, the generator intuitively performs the crucial operation of transforming raw partial point sets into clean and complete point sets. This model struggles to generate diverse samples, capture fine-grained details, or condition on sparse inputs. However, it can infer believable global structures [97]. Training GANs can be difficult due to common errors such as mode collapse [208]. This model, which focuses on object-level completion, works under the assumption that a single item has been found manually and that the input consists only of the points on this object. Consequently, this model is not appropriate for the goal of object detection [189].

**Figure 30:**
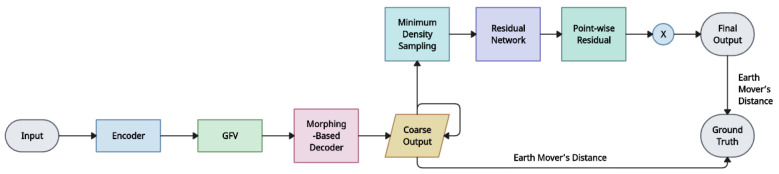
Architecture of MSN [209].

*Morphing and sampling-based network* [209]: A network that completes the partial point cloud in two steps has been proposed by the authors. Using an autoencoder architecture, a set of 2-manifold-like surface elements that can be 2D parameterised is used in the first stage to put together an entire point cloud. In order to obtain an evenly distributed subset point cloud from the combination of the coarse-grained prediction and the input point cloud, a sampling procedure is used in the second stage. Then, given the point cloud, a point-wise residual is learned, allowing for fine-grained features. This model uses the earth mover’s distance (EMD) as a better metric for measuring completion quality because, by solving the linear assignment problem, it forces model outputs to have the same density as the ground truths [210]. This model relieves the structural loss brought on by MLPs and recovers the entire point cloud of an object by estimating a group of parametric surface elements [211]. This approach frequently disregards the spatial correlation between points [190]. This model lacks the conditional generative ability based on partial observation, instead generating complete shapes mostly through learning a deterministic partial-to-complete mapping [212]. Although this approach produces encouraging results when applied to in-domain data, it is difficult to generalise to out-of-domain data, which includes real-world scans or data with various incomplete forms [213]. Figure 30 shows the architecture of the morphing and sampling-based network.

**Figure 31:**
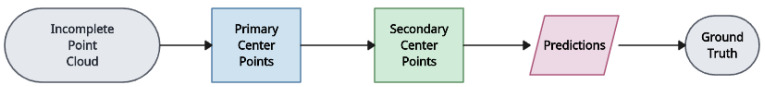
Architecture of PF-Net [214].

*PF-Net* [214]: This model accepts a partial point cloud as input and only outputs the portion of the point cloud that is missing, not the entire object, in order to maintain the original part’s spatial arrangements. As a result, it helps the network concentrate on identifying the location and structure of missing components by preserving the geometrical features of the original point cloud after restoration. Using a new feature extractor called combined multilayer perception (CMLP), the authors propose a multi-resolution encoder (MRE) to extract multilayer features from the partial point cloud and its low-resolution feature points. The missing point cloud is also intended to be generated hierarchically using a point pyramid decoder (PPD). PPD is a multi-scale generating network that predicts primary, secondary, and detailed points from layers with varying depths. It is based on feature points. The lack of utilisation of object completion and shape creation architectures, like PF-Net [214], by 3D object detectors during the LiDAR point cloud inference could result in improved detection performance [206]. This point cloud completion method’s max-pooling process during the encoding phase, where fine-grained information is lost and scarcely recoverable during the decoding phase, is its bottleneck [61]. In the ShapeNet-55 benchmarks, PFNet, which aims to predict objects’ missing components directly, fails because of the huge diversity [61]. This approach is still unable to predict a point splitting pattern that is locally structured. The primary issue is the fact that this approach solely concentrates on increasing the number of points and reconstructing the overall shape, neglecting to maintain an organised generation process for points inside specific regions. This makes it challenging to capture localised, intricate 3D shape structures and geometries using this method [190]. This model’s intricate design results in a comparatively large number of parameters [215]. Figure 31 shows the architecture of PF-Net.

**Figure 32:**
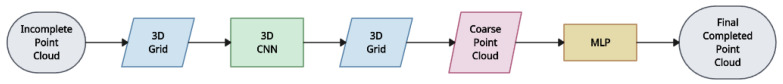
Overview of GRNet [211].

*GRNet* [211]: In order to regularise unordered point clouds and specifically maintain the structure and context of point clouds, the authors introduce 3D grids as intermediary representations. *Gridding*, *gridding reverse*, and *cubic feature sampling* are the three differentiable layers that make up the Gridding Residual Network (GRNet), which is proposed for point cloud completion together with 3D CNN and MLP. In the process of *gridding*, an interpolation function that quantifies the geometric relationships of the point cloud is used to weight the eight vertices of the 3D grid cell that each point in the point cloud resides in. The network then uses a 3D convolutional neural network (3D CNN) with skip connections to learn spatially and contextually aware features, filling in the gaps in the incomplete point cloud. *Gridding reverse* then replaces each 3D grid cell with a new point whose location is the weighted sum of the 3D grid cell’s eight vertices, converting the resulting 3D grid into a coarse point cloud. By concatenating the features of the corresponding eight vertices of the 3D grid cell that the point lies in; then, the following *cubic feature sampling* recovers features for every point in the coarse point cloud. To obtain the final finished point cloud, an MLP receives the features and the coarse point cloud. This model, which focuses on object-level completion, works under the assumption that a single item has been found manually and that the input consists only of the points on this object. Consequently, this model is not appropriate for the goal of object detection [189]. It is difficult to maintain a well-organised structure for points in small patches due to the discontinuous character of the point cloud and the unstructured prediction of points in local regions in this method [125]. Rebuilding low-resolution shapes is the only use for GRNet’s voxel representation. This model’s intricate design results in a comparatively large number of parameters [215]. Figure 32 shows the overview of GRNet.

**Figure 33:**
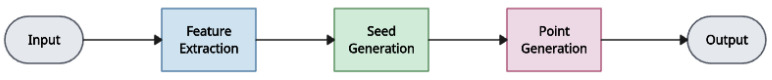
Overview of SnowflakeNet [190].

*SnowflakeNet* [190]: This model focuses specifically on the process of decoding incomplete point clouds. The primary building block of SnowflakeNet is its layers of snowflake point deconvolution (SPD), which simulate the creation of whole point clouds similar to the snowflake growth of points in three dimensions. This model creates points gradually by piling one SPD layer on top of another. Each SPD layer creates child points by dividing their parent point and inheriting the shape properties that the parent point captures. The purpose of disclosing geometrical details is to enable the use of a skip-transformer in SPD to identify point splitting modes that are most appropriate for specific localities. The current SPD layer is split by the skip-transformer, which uses an attention mechanism to summarise the splitting patterns from the previous SPD layer. The network is able to predict extremely detailed geometries because the locally compact, structured point cloud generated by SPD can precisely capture the structural properties of 3D shapes in limited patches. This model’s intricate design results in a comparatively large number of parameters [215]. Point clouds are sparse, thus recovering surfaces from them requires non-trivial postprocessing using traditional techniques [216]. There are two inherent limitations to the global feature structure that is extracted from partial inputs by this model. Firstly, fine-grained details are lost easily during pooling operations in the encoding phase and are difficult to recover from a diluted global feature during generation. Secondly, such a global feature is captured from a partial point cloud, which represents only the “incomplete” information of the visible part and goes against the goal of generating the complete shape [217]. Figure 33 shows the overview of SnowflakeNet.

**Table 13:** Advantages and limitations of point cloud completion models.

Model	Advantages	Limitations
PCN [205]	Acquires knowledge of a projection from the space of incomplete observations to the space of fully formed shapes.	Requires training data to be prepared in partial shapes since it expects a test input that is identical to the training data.
USCN [207]	Does not require explicit correspondence between example complete shape models and incomplete point sets.	Training GANs can be difficult due to common errors such as mode collapse.
MSN [209]	Uses EMD as a better metric for measuring completion quality.	Frequently disregards the spatial correlation between points.
PF-Net [214]	Accepts a partial point cloud as input and only outputs the portion of the point cloud that is missing.	Model’s intricate design results in a comparatively large number of parameters.
GRNet [211]	Uses 3D grids as intermediary representations to maintain unordered point clouds.	Difficult to maintain an organised structure for points in small patches due to the discontinuous character of the point cloud.
SnowflakeNet [190]	Focuses specifically on the process of decoding incomplete point clouds.	Fine-grained details are lost easily during pooling operations in the encoding phase.

## 8. Conclusions

This article presents a thorough examination of deep learning models applied in the areas of 3D reconstruction, registration, and augmentation. This study delivers an comprehensive overview of diverse models employed for these specific tasks. The advantages and disadvantages of the mentioned models are thoroughly analysed, highlighting the appropriateness of each approach for the specific task. In addition, the study analyses multiple datasets encompassing diverse activities and various 3D data formats. Deep learning has shown promising results in the areas of 3D registration, augmentation, and reconstruction. The objective of this survey was to examine the techniques used by deep learning frameworks for analysing and enhancing 3D image representation, augmentation, and reconstruction. The review of the literature thoroughly examined the advantages and disadvantages of different computer vision algorithms, network architectures, 3D structured data representation, and comparative data methodologies. Several point cloud completion techniques were also examined in relation to the advancement of deep-learning-based image processing technology.

Each phase of the generic methodology for 3D reconstruction, augmentation, and registration can be accomplished utilising distinct algorithms. Distinct methods are required for each constructed object, depending on its size, texture, and visual arrangement. In addition to efficient algorithms, the development of sensors has the potential to enhance the precision of 3D reconstruction in the future. Neural network modelling has numerous advantages. These operations are crucial for various sectors, including robots, autonomous autos, and medical imaging. The problem-solving precision and effectiveness of these domains have experienced a substantial improvement due to the efforts of deep learning models. In order to enhance the performance of these models, a significant amount of additional effort needs to be invested; the field is still in its early stages of development.

While there may be some who argue that traditional, non-deep-learning methods are more advantageous in certain situations, the review has demonstrated that deep learning models have consistently achieved state-of-the-art results in most cases. Given this evaluation, future research endeavours should prioritise the development of more accurate and efficient models capable of handling increasingly larger and more complex data. Moreover, the introduction of new datasets that more accurately represent real-life scenarios can help improve the effectiveness of these models. Furthermore, one attractive avenue for future research involves exploring the concatenation of diverse models to obtain enhanced outcomes.

## Figures and Tables

**Table 1:** 3D reconstruction models using point cloud data representation.

Model	Dataset	Data Representation
PointOutNet [9]	ShapeNet [10], 3D-R2N2 [11]	Point Cloud
Pseudo-renderer [12]	ShapeNet [10]	Point Cloud
RealPoint3D [13]	ShapeNet [10], ObjectNet3D [14]	Point Cloud
Cycle-consistency-based approach [15]	ShapeNet [10], Pix3D [16]	Point Cloud
3D34D [17]	ShapeNet [10]	Point Cloud
Unsupervised learning of 3D structure [18]	ShapeNet [10], MNIST3D [19]	Point Cloud

**Table 2:** 3D reconstruction models using voxel data representation.

Models	Dataset	Data Representation
GenRe [20]	ShapeNet [10], Pix3D [16]	Voxels
MarrNet [21]	ShapeNet [10], PASCAL3D+ [22]	Voxels
Perspective Transformer Nets [23]	ShapeNet [10]	Voxels
Rethinking reprojection [24]	ShapeNet [10], PASCAL3D+ [22], SUN [25], MS COCO [26]	Voxels
3D-GAN [27]	ModelNet [28], IKEA [29]	Voxels
Pix2Vox++ [30]	ShapeNet [10], Pix3D [16], Things3D [30]	Voxels
3D-R2N2 [11]	ShapeNet [10], PASCAL3D+ [22], MVS CAD 3D [11]	Voxels
Weak recon [31]	ShapeNet [10], ObjectNet3D [14]	Voxels
Relative viewpoint estimation [32]	ShapeNet [10], Pix3D [16], Things3D [30]	Voxels

**Table 4:** Benchmarking datasets included in this survey.

Datasets	Number of Frames	Number of Labels	Object Type	5 Common Classes
ModelNet [28]	151,128	660	3D CAD Scans	Bed, Chair, Desk, Sofa, Table
PASCAL3D+ [22]	30,899	12	3D CAD Scans	Boat, Bus, Car, Chair, Sofa
ShapeNet [10]	220,000	3135	Scans of Artefact, Plant, Person	Table, Car, Chair, Sofa, Rifle
ObjectNet3D [14]	90,127	100	Scans of Artifact, Vehicles	Bed, Car, Door, Fan, Key
ScanNet [39]	2,492,518	1513	Scans of Bedrooms, Kitchens, Offices	Bed, Chair, Door, Desk, Floor

**Table 5:** Single-view 3D reconstruction models reviewed in this study.

Nr.	Model	Dataset	Data Representation
1	PointOutNet [9]	ShapeNet [10], 3D-R2N2 [11]	Point Cloud
2	Pseudo-renderer [12]	ShapeNet [10]	Point Cloud
3	RealPoint3D [13]	ShapeNet [10], ObjectNet3D [14]	Point Cloud
4	Cycle- consistency-based [15] approach	ShapeNet [10], Pix3D [16]	Point Cloud
5	GenRe [20]	ShapeNet [10], Pix3D [16]	Voxels
6	MarrNet [21]	ShapeNet [10], PASCAL3D+ [22]	Voxels
7	Perspective Transformer [23] Nets	ShapeNet [10]	Voxels
8	Rethinking reprojection	ShapeNet [10], PASCAL3D+ [22], SUN [25], MS COCO [26]	Voxels
9	3D-GAN [24]	ModelNet [28], IKEA [29]	Voxels
10	Neural renderer [35]	ShapeNet [10]	Meshes
11	Residual MeshNet [36]	ShapeNet [10]	Meshes
12	Pixel2Mesh [37]	ShapeNet [10]	Meshes
13	CoReNet [38]	ShapeNet [10]	Meshes

**Table 6:** Advantages and limitations of single-view 3D reconstruction models.

Model	Advantages	Limitations
PointOutNet [9]	Introduces the chamfer distance loss, which is invariant to the permutation of points and is adopted by many other models as a regulariser.	Utilises less memory, but since they lack connection information, they need extensive postprocessing.
Pseudo-renderer [12]	Uses 2D supervision in addition to 3D supervision to obtain multiple projection images from various viewpoints of the generated 3D shape for optimisation.	Predicts denser, more accurate point clouds but is limited to the amount of points that point cloud-based representations can accommodate.
RealPoint3D [13]	Attempts to recreate 3D models from nature photographs with complicated backgrounds.	Needs an encoder to extract the input image’s 2D features and input point cloud data’s 3D features.
Cycle- consistency-based approach [15]	Uses a differentiable renderer to infer a 3D shape without using ground truth 3D annotation.	Cycle consistency produces deformed body structure or out-of-view images if it is unaware of the previous distribution of the 3D features, which interferes with the training process.
GenRe [20]	Can rebuild 3D objects with resolutions of up to 128 × 128 × 128 and more detailed reconstruction outcomes.	Higher resolutions have been used by this model at the expense of sluggish training or lossy 2D projections, as well as small training batches.
MarrNet [21]	Avoids modelling item appearance differences within the original image by generating 2.5D drawings from it.	Relies on 3D supervision which is only available for restricted classes or in a synthetic setting.
Perspective Transformer Nets [23]	Learns 3D volumetric representations from 2D observations based on principles of projective geometry.	Struggles to produce images that are consistent across several views as the underlying 3D scene structure cannot be utilised.
Rethinking reprojection [24]	Decoupling shape and posture lowers the number of free parameters in the network, increasing efficiency.	Assumes that the scene or object to be registered is either non-deformable or generally static.
3D-GAN [27]	Generative component aims to map a latent space to a distribution of intricate 3D shapes.	GAN training is notoriously unreliable.
Neural renderer [35]	Objects are trained in canonical pose.	This mesh renderer modifies geometry and colour in response to a target image.
Residual MeshNet [36]	Reconstructing 3D meshes using MLPs in a cascaded hierarchical fashion.	Produces mesh automatically during the finite element method (FEM) computation process, although it does not save time increasing computing productivity.
Pixel2Mesh [37]	Extracts perceptual features from the input image and gradually deforms an ellipsoid in order to obtain the output geometry.	Several perspectives of the target object or scene are not included in the training data for 3D shape reconstruction, as in real-world scenarios.
CoReNet [38]	Reconstructs the shape and semantic class of many objects directly in a 3D volumetric grid using a single RGB image.	Training on synthetic representations restricts their practicality in real-world situations.

**Table 7:** Multiple-view 3D reconstruction models reviewed in this study.

Nr.	Model	Dataset	Data Representation
1	3D34D [17]	ShapeNet [10]	Point Cloud
2	Unsupervised learning of 3D structure [18]	ShapeNet [10], MNIST3D [19]	Point Cloud
3	Pix2Vox++ [30]	ShapeNet [10], Pix3D [16], Things3D [30]	Voxels
4	3D-R2N2 [11]	ShapeNet [10], PASCAL3D+ [22], MVS CAD 3D [11]	Voxels
5	Weak recon [31]	ShapeNet [10], ObjectNet3D [14]	Voxels
6	Relative viewpoint estimation [32]	ShapeNet [10], Pix3D [16], Things3D [30]	Voxels

**Table 8:** Advantages and limitations of multi-view 3D reconstruction models.

Model	Advantages	Limitations
3D34D [17]	Obtains a more expressive intermediate shape representation by locally assigning features and 3D points.	Performs admirably on synthetic objects rendered with a clear background, but not on actual photos, novel categories, or more intricate object geometries.
Unsupervised learning of 3D structures [18]	Optimises 3D representations to provide realistic 2D images from all randomly sampled views.	Only basic and coarse shapes can be reconstructed.
Pix2Vox++ [30]	Generates a coarse volume for each input image.	Because of memory limitations, the model’s cubic complexity in space results in coarse discretisations.
3D-R2N2 [11]	Converts RGB image partial inputs into a latent vector, which is then used to predict the complete volumetric shape using previously learned priors.	Only works with coarse 64 × 64 × 64 grids.
Weak recon [31]	Alternative to costly 3D CAD annotation, and proposes using lower-cost 2D supervision.	Reconstructions are hampered by this weakly supervised environment.
Relative viewpoint estimation [32]	Predicts a transformation that optimally matches the bottleneck features of two input images during testing.	It can only predict posture for instances of a single item and does not extend to the category level.

**Table 9:** 3D registration models reviewed in this study.

Nr.	Model	Dataset	Data Representation
1	CPD [136]	Stanford Bunny [33]	Meshes
2	PSR-SDP [137]	TUM RGB-D [138]	Point Cloud
3	RPM-Net [139]	ModelNet [28]	Meshes
4	DeepICP [140]	KITTI [141], SouthBay [142]	Point Cloud, Voxels
5	3D-SmoothNet [143]	3DMatch [144]	Point Cloud, Voxels
6	3D multi-view registration [145]	3DMatch [144], Redwood [146], ScanNet [39]	Point Cloud

**Table 10:** Advantages and limitations of 3D registration models.

Model	Advantages	Limitations
CPD [136]	Considers the alignment as a probability density estimation problem, where one point cloud set represents the Gaussian mixture model centroids, and the other represents the data points.	While GMM-based methods might increase resilience against outliers and bad initialisations, local search remains the foundation of the optimisation.
PSR-SDP [137]	Allows for verifying the global optimality of a local minimiser in a significantly faster manner.	Provides poor estimates even in the presence of a single outlier because it assumes that all measurements are inliers.
RPM-Net [139]	Able to solve the partial visibility of the point cloud and obtain a soft assignment of point correspondences.	Computational efficacy increases as the number of points in the point clouds increases.
DeepICP [140]	By creating a connection using the point cloud’s learned attributes, this study improved the conventional ICP algorithm using the neural network technique.	Takes a lot of computing effort to combine deep learning with ICP directly.
3DSmoothNet [143]	First learned, universal matching method that allows transferring trained models between modalities.	290 times slower than FCGF [162] model.
3D multi-view registration [145]	First end-to-end algorithm for joint learning of both stages of the registration problem.	A lot of training data are required.

**Table 11:** 3D augmentation models reviewed in this study.

Nr.	Model	Dataset	Data Representation
1	MaskNet [165]	S3DIS [3], 3DMatch [144], ModelNet [28]	Point Cloud
2	GPDNet [166]	ShapeNet [10]	Point Cloud
3	DMR [167]	ModelNet [28]	Point Cloud
4	PU-Net [168]	ModelNet [28], ShapeNet [10]	Point Cloud
5	MPU [169]	ModelNet [28], MNIST-CP [19]	Point Cloud
6	CP-Net [170]	ModelNet [28]	Point Cloud
7	SampleNet [171]	ModelNet [28], ShapeNet [10]	Point Cloud

**Table 12:** Advantages and limitations of 3D augmentation models.

Model	Advantages	Limitations
MaskNet [165]	Rejects noise in even partial clouds in a rather computationally inexpensive manner.	Requires the input of both a partial and complete point cloud.
GDPNet [166]	Deals with the permutation-invariance problem and builds hierarchies of local or non-local features to effectively address the denoising problem.	The point clouds’ geometric characteristics are often oversmoothed.
DMR [167]	Patch manifold reconstruction (PMR) upsampling technique is straightforward and efficient.	Downsampling step invariably results in detail loss, especially at low noise levels, and could also oversmooth by removing some useful information.
PU-Net [168]	Both reconstruction loss and repulsion loss are jointly utilised to improve the quality of the output.	Only learns spatial relationships at a single level of multi-step point cloud decoding via self-attention.
MPU [169]	Trained end-to-end on high-resolution point clouds and emphasises a certain level of detail by altering the spatial span of the receptive field in various steps.	Cannot be used for completion tasks and is restricted to upsampling sparse locations.
CP-Net [170]	Final representations typically retain crucial points that take up a significant number of channels.	Potential loss of information due to the down-sampling process.
SampleNet [171]	Sampling procedure for the representative point cloud classification problem becomes differentiable, allowing for end-to-end optimisation.	Fails to attain a satisfactory equilibrium between maintaining geometric features and uniform density.

## Data Availability

Not applicable.

## Abbreviations

The following abbreviations are used in this manuscript:

3D	Three-dimensional
2D	Two-dimensional
LiDAR	Light detection and ranging
RGB-D	Red, green, blue plus depth
CAD	Computer-aided design
MLP	Multilayer perceptron
CNN	Convolutional neural network
FCGFs	Fully convolutional geometric features
GPU	Graphics processing unit
RAM	Random access memory
